# Dissection of the *cis*-2-decenoic acid signaling network in *Pseudomonas aeruginosa* using microarray technique

**DOI:** 10.3389/fmicb.2015.00383

**Published:** 2015-04-28

**Authors:** Azadeh Rahmani-Badi, Shayesteh Sepehr, Hossein Fallahi, Saeed Heidari-Keshel

**Affiliations:** ^1^Department of Biology, School of Science, Alzahra UniversityTehran, Iran; ^2^Department of Biology, School of Science, Razi UniversityKermanshah, Iran; ^3^Stem Cell Preparation Unit, Eye Research Center, Farabi Eye HospitalTehran, Iran

**Keywords:** *cis*-2-decenoic acid signaling, dispersal, microarray experiment, protein-protein interaction (PPI), signal transduction, virulence

## Abstract

Many bacterial pathogens use quorum-sensing (QS) signaling to regulate the expression of factors contributing to virulence and persistence. Bacteria produce signals of different chemical classes. The signal molecule, known as diffusible signal factor (DSF), is a *cis*-unsaturated fatty acid that was first described in the plant pathogen *Xanthomonas campestris*. Previous works have shown that human pathogen, *Pseudomonas aeruginosa*, also synthesizes a structurally related molecule, characterized as *cis-2-decenoic* acid (C_10_: Δ^2^, CDA) that induces biofilm dispersal by multiple types of bacteria. Furthermore, CDA has been shown to be involved in inter-kingdom signaling that modulates fungal behavior. Therefore, an understanding of its signaling mechanism could suggest strategies for interference, with consequences for disease control. To identify the components of CDA signaling pathway in this pathogen, a comparative transcritpome analysis was conducted, in the presence and absence of CDA. A protein-protein interaction (PPI) network for differentially expressed (DE) genes with known function was then constructed by STRING and Cytoscape. In addition, the effects of CDA in combination with antimicrobial agents on the biofilm surface area and bacteria viability were evaluated using fluorescence microscopy and digital image analysis. Microarray analysis identified 666 differentially expressed genes in the presence of CDA and gene ontology (GO) analysis revealed that in *P. aeruginosa*, CDA mediates dispersion of biofilms through signaling pathways, including enhanced motility, metabolic activity, virulence as well as persistence at different temperatures. PPI data suggested that a cluster of five genes (*PA4978*, *PA4979*, *PA4980*, *PA4982*, *PA4983*) is involved in the CDA synthesis and perception. Combined treatments using both CDA and antimicrobial agents showed that following exposure of the biofilms to CDA, remaining cells on the surface were easily removed and killed by antimicrobials.

## Introduction

Intercellular signaling regulates the persistence and virulence of many pathogenic bacteria (McCarthy et al., 2010). These bacteria produce chemically different signal molecules to monitor their environment such as cell density, known as quorum sensing (QS), or confinement to niches and to modulate own behavior consequently (McCarthy et al., 2010; Ryan and Dow, 2011). Therefore, interfering with these signaling pathways presents rational approaches to treat infections (McCarthy et al., 2010; Ryan and Dow, 2011). However, such approaches require an understanding of both the mechanisms of signaling and their role in behavior of bacteria (McCarthy et al., 2010). The QS signaling network of *Pseudomonas aeruginosa*, a gram-negative ubiquitous bacterium, is one of the most complicated QS systems in all bacterial species (Jimenez et al., 2012; Liang et al., 2014). It consists of multiple interconnected signaling layers that coordinately regulate virulence and persistence (Jimenez et al., 2012).

*P. aeruginosa* is an important opportunistic human pathogen linked to chronic colonization of a wide range of human tissues and medical devices (Petrova and Sauer, 2010). This pathogen is considered one of the primary causes of persistent infections in immune-compromised hosts and also is the main etiologic cause of morbidity and mortality in cystic fibrosis (CF) (Van Delden and Iglewski, 1998; Hutchison and Govan, 1999; Gibson et al., 2003; Musk and Hergenrother, 2008). Treatment of its infections is complicated due to the ability of this organism to form communities, attached to surfaces known as biofilms (Musk and Hergenrother, 2008; Goldová et al., 2011; Schluter et al., 2015). Biofilms contain differentiated bacteria that possess enhanced antibiotic resistance ability, compared to their planktonic counterparts (Barraud et al., 2006; Goldová et al., 2011). Biofilm formation in *P. aeruginosa* is governed by regulation of different genes involved in motility, adhesion, and exo-polysaccharide (EPS) synthesis pathways in response to inter- and intracellular signaling molecules and environmental cues (Petrova and Sauer, 2009; Coggan and Wolfgang, 2012). A deep understanding of dispersal regulation, including the role of signals, offers promise for development of novel approaches to control *P. aeruginosa* infections. In this pathogen induction of biofilm dispersal naturally happens when microcolonies within biofilms reach a critical size, releasing bacteria as free-swimming cells into the surrounding environment (Davies and Marques, 2009; Amari et al., 2013). Recently, Davies and Marques demonstrated that the small fatty acid molecule *cis*-2-decenoic acid (C_10_: Δ^2^, CDA), produced by *P. aeruginosa* both in batch and continuous cultures, acts as the autoinducer of biofilm dispersion for this pathogen (Davies and Marques, 2009; Amari et al., 2013). CDA also induces the dispersion of established biofilms formed by multiple types of bacteria as well as *Candida albicans*, suggesting an inter-species and inter-kingdom role for this molecule (Davies and Marques, 2009; Rahmani-Badi et al., 2014; Sepehr et al., 2014). The autoinducer CDA is a fatty acid cell-to-cell communication molecule with structural homology to *cis*-11-methyl-2-dodecenoic acid (DSF), isolated from plant pathogen *Xanthomonas campestris* pv. *campestris* (*Xcc*) (Davies and Marques, 2009; Amari et al., 2013). While comparative genomic analysis has shown that DSF pathway regulates diverse biological functions including biofilm dispersal, virulence, and ecological competence in *Xcc*, a little is known about the CDA signaling network and the regulated functions in *P. aeruginosa* (He et al., 2006). Synthesis and detection of DSF require products of the *rpf* (for regulation of pathogenicity factors) gene cluster (Davies and Marques, 2009). Synthesis of DSF involves RpfF, an enoyl coenzyme A (CoA) hydratase and RpfB, a long-chain fatty acyl CoA ligase (Dow et al., 2003; He et al., 2006; Davies and Marques, 2009; Ryan and Dow, 2011), whereas DSF perception requires a two-component signal transduction system, including RpfC, a sensor kinase and RpfG, a response regulator (Dow et al., 2003; He et al., 2006; Davies and Marques, 2009; Ryan and Dow, 2011). Although CDA and DSF are structurally similar molecules, bioinformatics analysis does not disclose the presence of an *rpf* gene cluster in *P. aeruginosa*.

Therefore, in this study to identify the components of the CDA signaling pathway, a comparative microarray analysis of the transcriptome, in the presence and absence of CDA, was conducted for *P. aeruginosa* biofilms. Then, to identify the cellular processes which are linked together and are expressed in the presence of CDA, a protein-protein interaction (PPI) network for differentially expressed (DE) genes with known function was constructed by STRING (Search Tool for the Retrieval of Interacting Genes) database (Szklarczyk et al., 2011) and Cytoscape software (Shannon et al., 2003). Besides, the efficiency of two broadly used antibiotics (tobramycin and ciprofloxacin) as well as hydrogen peroxide (H_2_O_2_) alone or in combination with CDA to remove *P. aeruginosa* established biofilms was examined.

## Materials and methods

### Bacterial strains and growth conditions

*P. aeruginosa* strain PAO1 (ATCC 15692) was generously provided by Mehri Michea-Hamzeh Pour (Department of Genetics and Microbiology, Center Medical University, CH-1211 Genova 4, Switzerland). Overnight cultures of *P. aeruginosa* PAO1 were routinely grown in Luria-Bertani (LB) medium (Merck) at 37°C with continuous shaking (Barraud et al., 2006). Biofilms were grown in modified M9 minimal medium (Webb et al., 2003) with glucose at 5 mM for continuous-culture experiments and 20 mM for semi-batch culture petri dish experiments (Barraud et al., 2006).

### Biofilm growth and dispersal in semi-batch culture

*P. aeruginosa* PAO1 overnight cultures were diluted 1:1000 into fifteen ml of growth medium, inoculated in sterile petri dishes and incubated at room temperature with 30 rpm shaking (Rahmani-Badi et al., 2014; Sepehr et al., 2014). Medium in the plates was replaced every 24 h for 5 days to allow the accumulation of sufficient biofilm biomass, while reducing the native dispersion due to the build-up of endogenous inducer (Davies and Marques, 2009; Rahmani-Badi et al., 2014; Sepehr et al., 2014). After the last exchange of medium, the cells were allowed to grow for about 1 h and then dispersion induction was tested by replacing the growth medium with fresh medium containing different concentrations of commercially synthesized CDA (50, 100, 310, and 620 nM) or just the carrier (10% ethanol) as a control and the cells were incubated for a further 1 h (Rahmani-Badi et al., 2014; Sepehr et al., 2014). Medium containing dispersed cells was then transferred by pipette to a 50 ml Erlenmeyer and was subsequently homogenized for 30 s at 5000 rpm with a WiseTis-Homogenizer model HG-150 (Daihan Scientific Co., Ltd., Korea) to ensure the separation of cells (Sepehr et al., 2014). The cell density was determined based on the OD_600_ with an UV/VIS spectrophotometer model T80^+^ (PG Instruments, Ltd., China) (Sepehr et al., 2014). Biofilm dispersal bioassays were performed in triplicates in at least four individual experiments for each concentration.

### Biofilm growth and dispersal in flow cell

To observe the effect of CDA on biofilm dispersal, biofilms were also grown in continuous culture flow cells. The flow cell (channel dimensions, 1 × 4 × 40 mm) was constructed of polycarbonate and capped with a glass cover slip (Sepehr et al., 2014). Sterile M9 medium was pumped from a 5-Liter vessel through silicone tubing (1 mm in diameter) to the flow cell using an eight-roller-head peristaltic pump (Baoding Longer Precision Pump Co., Ltd., China) at a flow rate of 280 μl/min (Sepehr et al., 2014). Medium leaving the flow cell was discharged to an effluent reservoir via silicone tubing (1 mm in diameter). The entire system was closed to the outside environment but maintained in equilibrium with atmospheric pressure by a 0.2-μm-pore-size gas-permeable filter fitted to each vessel (Davies and Marques, 2009; Sepehr et al., 2014). Channels were inoculated with *P. aeruginosa* PAO1 overnight cultures diluted 1:1000 in growth medium and flow was initiated after 1 h at an elution rate of 280 μl/min (Sepehr et al., 2014). After 48 h of biofilm cultures, the influent medium was switched from fresh medium in the test lines to one of the indicated concentrations of CDA and control lines were switched to new lines containing only carrier (Sepehr et al., 2014). Standard silicone tubing was replaced with autoclaved glass tubing for the collection of flow cell effluent runoff and after 1 h treatment the number of dispersed cells was determined based on the OD_600_. The biofilms remaining on the surface of coverslip were then stained with 3 μl/ml SYTO 9 (Molecular Probes, Invitrogen). Using epifluorescence microscopy (CETI, Belgium), 15 selected fields of view per flow cell were imaged in the XY plane, at regular intervals and across the entire channels (Barraud et al., 2009a; Rahmani-Badi et al., 2014; Sepehr et al., 2014). Image analysis was performed using ImageJ Software (NIH) with automatic threshold definition and results were presented as the percentage of total biofilm surface reduction in cultures treated with different concentrations of CDA relative to the total biofilm surface in control cultures that were not exposed to CDA (Barraud et al., 2009a; Rahmani-Badi et al., 2014; Sepehr et al., 2014). Three replicates per experiment were used and at least 2 independent repetitions of experiments were performed.

### RNA extraction and microarray hybridization

*P. aeruginosa* PAO1 biofilms were cultivated on the interior surfaces of tubing of a once-through continuous-flow-reactor system at room temperature (25°C) with a flow rate of 280 μl/min (Rahmani-Badi et al., 2014; Sepehr et al., 2014). After 5 days, established biofilms were exposed to 100 nM CDA or left untreated (as a control). This concentration of CDA while causing a significant dispersal in biofilms (*P* < 0.05) still allowed sufficient biofilm biomass remaining after induction of dispersal to be harvested for subsequent RNA extraction. After 1 h, the biofilms remaining on the walls of the tubing were washed by a gentle bath of phosphate buffer saline (PBS) to remove planktonic bacteria and biofilm cells were immediately re-suspended in RNAprotect Bacteria Reagent (Qiagen) by pressing/squeezing the tubing firmly by hand, throughout the length of the tubing. Total RNA was then extracted using RNeasy mini kit (Qiagen). Purified total RNA was treated with deoxyribonuclease I (Thermo scientific). RNA preparations were checked for DNA contamination by PCR and quantified spectrometrically at 260 and 280 nm. RNA quality was further checked by formaldehyde agarose gel electrophoresis. cDNA was synthesized and aminoallyl labeled using Aminoallyl labeling kit for DNA microarrays (Arrayit). After cDNA purification, labeling was performed using CyDye post-labeling reactive dye pack (Amersham, GE Healthcare). Labeling efficiency was then assessed using a NanoDrop ND1000 spectrophotometer (NanoDrop Technologies, Wilmington) and samples were hybridized using hybridization station TrayMix S4 (Biotray) at 42°C for 5 h.

### Microarray analysis

*P. aeruginosa* PAO1 glass slide arrays (Mycroarray Inc.) were scanned using a ScanArray Express scanner (Perkin-Elmer). Raw data (spots intensity) were obtained by image processing through ProScanArray Express (Version 4.0) software. Data normalization and calibration were conducted by applying Robust Multichip Averaging (RMA) algorithm (Irizarry et al., 2003) in R package (Gentleman et al., 2004). Data are deposited in the Gene Expression Omnibus (GEO) database under accession number GSE57594. Identification of differentially expressed genes conducted using Flexarray software (Blazejczyk, 2007). Known PPI data with confidence score larger than 0.8 for DE genes with known function were obtained from STRING 9.1 database (Szklarczyk et al., 2011). Cytoscape software v.3 (Shannon et al., 2003) was then applied to visualize these interactions, following use of *P. aeruginosa* database to convert the gene name to an official gene symbol (locus tags). Finally, expression data were loaded on the network. Cluster 3.0 (Eisen et al., 1998; de Hoon et al., 2004) was used for clustering the genes based on their fold changes by applying a hierarchical algorithm as statistical method for clustering and result visualized using Java TreeViewer (Saldanha, 2004).

### Semi-quantitative reverse transcription-polymerase chain reaction (RT-PCR)

To validate observed changes in gene expression in the microarray experiments, the mRNA levels of five genes (*algD, katA, flhA, phoP*, and *pvdS*) and one housekeeping gene (*proC*) from biofilm cells treated with or without 100 nM CDA were examined by semi-quantitative RT-PCR, using the same experimental conditions as those for transcriptomic analysis were assessed. Primers for each selected gene were designed (Supplemental Table 1) and checked using www.ncbi.nlm.nih.gov/tools/primer-blast website. RT-PCR was carried out in a total volume of 50 μl, consisting of 25 μl 2X Vivantis PCR Master Mix (USA) and 4 μl cDNA. The final concentrations of Mg^+2^ and primers were respectively 2.5 mM and 50 pmol per reaction. PCR was accomplished after a 5 min activation and denaturation step at 95°C, followed by 45 cycles of 20 s at 95°C, 30 s at 60°C, 15 s at 72°C and final extension 5 min at 72°C. Negative controls containing RNA instead of cDNA were run concomitantly to confirm that samples were free from contamination. Images of the RT-PCR ethidium bromide-stained agarose gels were acquired with a Sony XC-75 CE camera (Vilber Lourmat Inc. Cedex, France) and quantification of the bands was performed by ImageJ software (NIH). Band intensity was expressed as relative absorbance units (Marone et al., 2001). The ratio between the sample RNA to be determined and *proC* was calculated to normalize for initial variations in sample concentration and as a control for reaction efficiency (Marone et al., 2001). Mean and standard deviation of all experiments performed were calculated after normalization to *proC* (Marone et al., 2001).

### CDA combined treatments of *P. aeruginosa* biofilms and planktonic cells

Flow cells were inoculated with PAO1overnight cultures diluted 1:1000 in M9 medium and flow was initiated after 1 h at an elution rate of 280 μl/min (Rahmani-Badi et al., 2014; Sepehr et al., 2014). After 48 h of biofilm cultures, the influent medium was switched from fresh medium in the test lines to one of the antibiotics (tobramycin (Sigma) at a final concentration of 64 μg/ml, ciprofloxacin (Sigma) at 1 μg/ml) or hydrogen peroxide (H_2_O_2_) (Merck) at 100 ppm, alone or in combination with 100 nM CDA. The concentrations of antibiotics and disinfectant selected for use were previously established to be effective against planktonic cells but have no inhibitory effect on the *P. aeruginosa* biofilm cells (Barraud et al., 2006). Control lines were switched to new lines containing only carrier. Following 1 h treatment, biofilms were stained with a LIVE/DEAD *Bac*Light bacterial viability kit (Molecular Probes, Invitrogen). Live SYTO9-stained cells and dead propidium iodide-stained cells were visualized using epifluorescence microscopy (CETI, Belgium). Fifteen selected fields of view per flow cell were imaged in the XY plane, at regular intervals and across the entire channels (Barraud et al., 2009a; Rahmani-Badi et al., 2014; Sepehr et al., 2014). Image analysis was performed using ImageJ Software (NIH) with automatic threshold definition and results were presented as the percentage of total biofilm surface reduction in cultures treated with different concentrations of CDA relative to the total biofilm surface in control cultures that were not exposed to CDA.

Three replicates per experiment were used and at least two independent repetition experiments were performed.

We have also evaluated the probable inhibitory effects of antibiotics and the disinfectant, H_2_O_2_, alone or in combination with 100 nM CDA on the growth of planktonic cells of *P. aeruginosa*. The MICs were determined in triplicate in Mueller-Hinton broth using micro-dilution assay with bacteria at a density of 10^5^CFU/ml. Plates were incubated for 24 h at 37°C. The MIC was selected as the lowest concentration of antibiotics or disinfectant where there was no growth after 24 h (Clinical and Laboratory Standards Institute, 2006).

### Statistical analysis

All data were analyzed using analysis of variance (ANOVA) by the general linear model procedure of Minitab data analysis software (release 16, Minitab Inc., PA. USA). Pairwise comparisons were then made between all of the groups using Tukey's method. *P* < 0.05 were regarded as significant. All measurements were carried out in triplicate.

## Results

### CDA caused a concentration-dependent increase in the population of planktonic cells

**Figure 1:**
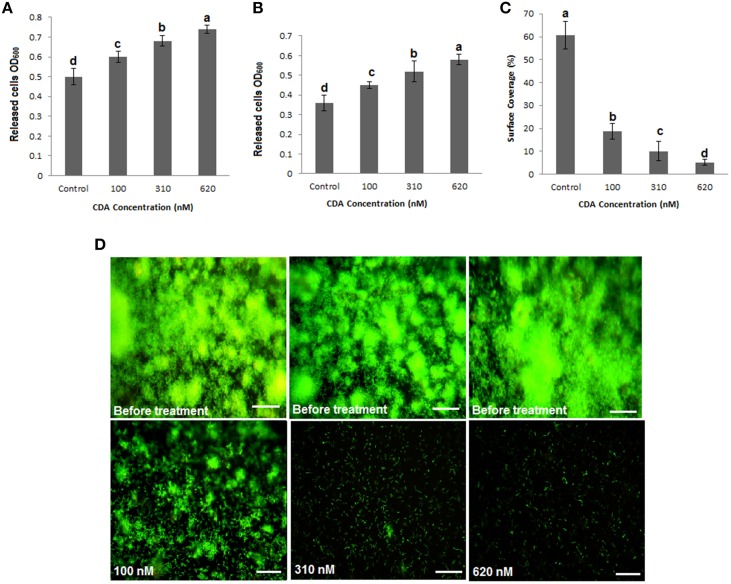
**Induction of planktonic mode of growth in *P. aeruginosa* PAO1 biofilms by CDA. (A)** Determination of cell density using semi-batch cultures. *P. aeruginosa* biofilms were grown for 5 days in petri dishes. Dispersion by CDA was tested at concentrations between 100 and 620 nM (carrier used as a control). Cell density was determined by measuring the optical density. **(B)** Determination of cell density using continuous cultures. Biofilms of *P. aeruginosa* PAO1 were grown in flow cell continuous cultures, and dispersed by given CDA concentrations or the carrier. Effluent runoffs were then collected and cell density was determined. **(C)** Quantification of percent surface coverage. *P. aeruginosa* PAO1 biofilms remaining on the surface were then stained with 3 μl/ml SYTO 9 to allow analysis using fluorescence microscopy and percent surface coverage was quantified using digital image analysis. Error bars indicate SD (*n* = 3). **(D)** Stained biofilms before and after treatments by CDA. Images are top-down views (x-y plane); scale bars: 50 μm.

It was previously demonstrated that CDA induces dispersal in *P. aeruginosa* biofilms (Davies and Marques, 2009). While its native concentration in the supernatant of *P. aeruginosa* cultures has been reported to be 2.5 nM, its final concentration (as an inducer of biofilm dispersal) should be between 10 and 620 nM, when added exogenously (Davies and Marques, 2009). Our studies showed that concentrations of CDA below 100 nM did not induce a significant dispersal in *P. aeruginosa* biofilms that were grown in two different systems, both semi-batch (petri dishes) and continuous (flow cells). Therefore, we tested higher concentrations of CDA, including 100, 310, and 620 nM to assess their effects on the *P. aeruginosa* PAO1 biofilm biomass. CDA treatments caused a concentration- dependent increase in the population of planktonic cells released into the bulk liquid of petri dishes or in the effluent runoffs of flow cells compared to untreated controls (*P* < 0.05). Consequently, when established biofilms, cultivated in petri dishes or flow cells, were exposed to CDA for only 1 h, we observed that all tested concentrations of CDA reversed biofilm formation and caused detachment and dispersal of cells from the surface (Figure 1). The highest increase in the planktonic biomass was observed when both semi-batch and continuous cultures were treated with 620 nM CDA (OD_600_ = 0.72 ± 0.02, SD, *P* < 0.05 for petri dish and OD_600_ = 0.56 ± 0.02, SD, *P* < 0.05 for flow cell dispersal bioassays, respectively) compared to results for untreated controls (OD_600_ = 0.5 ± 0.04, SD, *P* < 0.05 for petri dish and OD_600_ = 0.36 ± 0.04, SD, *P* < 0.05 for flow cell cultures, respectively) (Figures 1A,B). Whereas, treatment of the biofilms with 100 nM CDA resulted in 55% reduction in biofilm surface coverage (Figure 1C), after treatment of biofilms with 620 nM CDA only a few cells remained attached to the cover slip (Figure 1D). Therefore, to collect enough cells for mRNAs extraction, we used 100 nM CDA treatments for the gene expression analysis.

### CDA modulates expression of a wide range of genes involved in various functions

**Figure 2:**
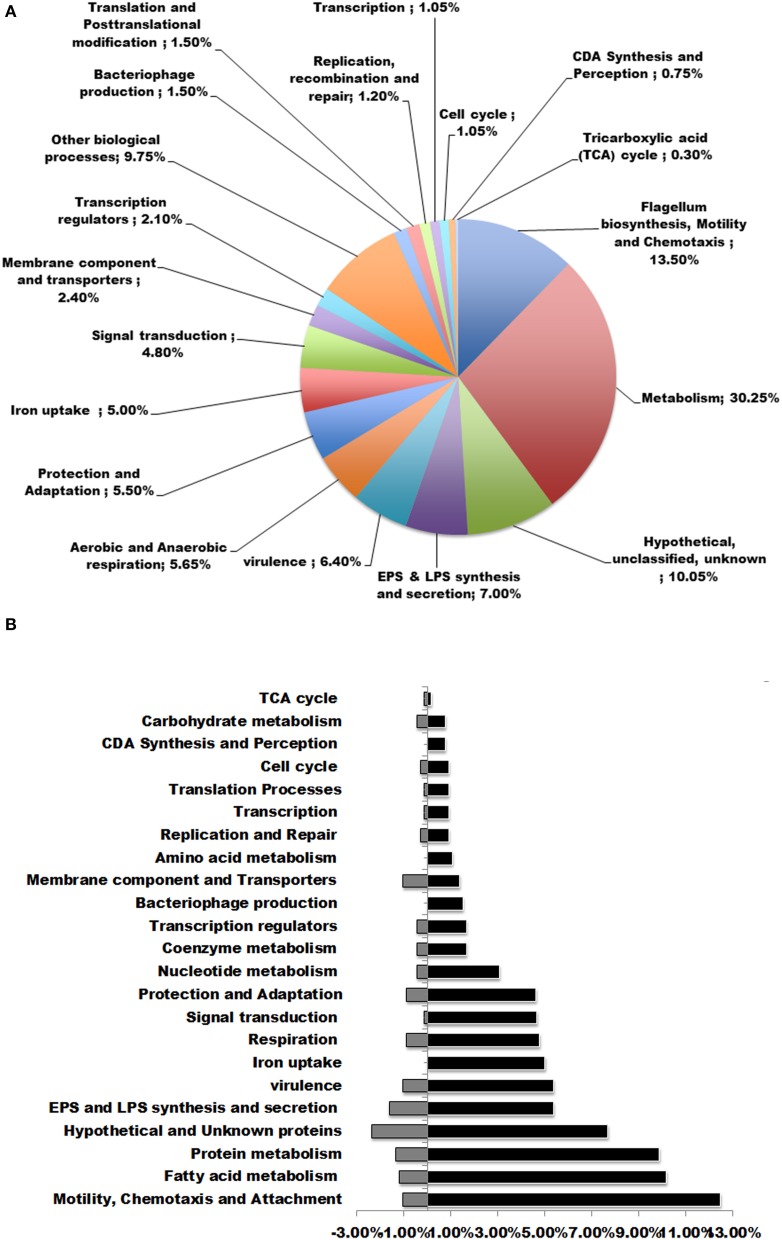
**Functional classifications of DE genes in *P. aeruginosa* after treatment with 100 nM CDA. (A)** DE genes were classified in to more than 15 functional groups. The top functional classes with the percentages of genes altered in each class were presented in the pie graph. **(B)** Percentages of up and down regulated genes in each functional group.

To investigate the transcriptome profile alterations caused by CDA treatment, we compared the gene expression patterns of 5-day-old biofilms of *P. aerugionsa* PAO1 in the presence and absence of 100 nM CDA. The comparative analysis of DE genes led us to identify 666 CDA-regulated genes that were randomly scattered throughout the bacterial genome. Most of the genes, 523 of the 666 genes (91%), showed up regulation, while the remaining 143 genes (8.9%) were down regulated in the presence of CDA (Supplemental Table 2, Table S3). Functional classification of these DE genes revealed that, except for 64 genes encoding hypothetical proteins with unknown functions, the products of the remaining 607 CDA-responsive genes could be classified into the following biological functions (Table 1, Figure 2A): synthesis, sensing and responding to CDA (*PA4980, PA4982-PA4983*), flagellum synthesis (e.g., *fliCDFGJMNOQR, flgBCDEGHI, flhAB)*, type IV fimbrial biosynthesis (e.g., *pilDEMPRSUZ)*, chemotaxis (e.g., *cheYZ, wspABCEFR)*, attachment (e.g., *cupA3A4B2B5*), resistance to oxidative stresses (e.g., *sodBM, trxA, katAB, ahpCF*), persistence at different temperatures (e.g., *hscA, hslV*, *PA0456, PA0961*), iron uptake (e.g., *foxAR, fpvAR, prpL, toxR, rsaL, pfeR*), lipopolysaccharide (LPS) synthesis and secretion (e.g., *waaACFG, wbpMWZ, rfaDE, lpxDO2)*, extracellular polysaccharide synthesis and secretion (e.g., *algABEFIJLQRUWXZ*), virulence (e.g., *toxA, antAB, aprA, phzD1D2M*), bacteriophage production (e.g., *PA0617-PA0623*), transcription regulators (e.g., *rpoN*), aerobic and anaerobic respiration (e.g., *aer*, *nirBCDFJLMNQS*, *narHIJK1K2*), tri-carboxylic acid (TCA) cycle (e.g., *acnA*), signal transduction mechanisms (e.g., *pprAB, mifS, bfiRS*), metabolism of fatty acids (e.g., *fabABDF1F2GH2IZ*), amino acids (e.g., *pauA3A5*), nucleic acids (e.g., *purHLMN, pyrDEFH*) and carbohydrates (e.g., *pykF*), membrane components and transporters (e.g., *pstAB, opdH*), post-translational modification (e.g., *dnaK, clpBXPP2*) and others. Figure 2B shows the percentage of up and down regulated genes in each functional group.

**Table 1:** Functional classification of CDA-regulated genes obtained from DE gene list^*^.

**Gene family**	**Gene name or ID**
Synthesis, sensing, and responding to CDA	*PA4978-PA4980, PA4982-PA4983*
Flagellum and Type IV fimbrial biosynthesis, attachment, motility, and chemotaxis	*fliCDFGJMNOQR, flhAB, fppA, fimX, tadCDGZ, flgBCDEGHI, motABC, fleQR, cheYZ, migA, ntrB, pilDEMOPRSUZ, pctAC, aer2, wspABCEFR, phoB, phoU, phoR, cupA3A4B2B5, PA0173-PA0178, PA1095, PA1103, PA1442, PA1458 -PA1459, PA1463-PA1464, PA1608, PA1930, PA2561, PA2573, PA2652, PA2654, PA2867, PA3348, PA4290, PA4300, PA4332, PA4457, PA4520, PA4633, PA4844, PA4915, PA5072*
Iron uptake	*pvdADEFGJLNOPS, foxAR, fpvAR, prpL, toxR, rsaL, pfeR, pchCDFG, pprB, pcpS, PA0434-PA0435, PA0615, PA2402, PA2465, PA2468, PA4467, PA4471*
Fatty acid metabolism and transport	*plsX, tktA, pgsA, gapA, glpD, lptA, hisB, ribB, faoA, pgk, gnyBH, accD, desB, fadD1, fabABDF1F2GH2IZ, uppS, mdcA, atuC,PA0098, PA0182,PA0286, PA0493, PA0506-PA0508, PA0745-PA0746, PA0879, PA1020-PA1022, PA1187, PA1240, PA1470, PA1535, PA1576, PA1628-PA1629, PA1631, PA1827, PA1869, PA2550, PA2552, PA2815, PA2841, PA2887-PA2891, PA2893, PA3286, PA3426, PA3589, PA3591, PA3593, PA3924,PA4089, PA4330, PA4912, PA4979-PA4980, PA4995, PA5020, PA5524*
Protein and Amino acid metabolism	*thrS, folC, glnA, gmk, tgt, dadA, pauA3A5, gltX, gcvT1T2, glyQ, gdhA, valS, purD, trmD, speA, metGK, hutH, lysC, gpuA, mdlC, glnS, ansB, ileS, rpsBGILP, frr, groES, groEL, urea, lipB, sahH, betA, aspA, ilvCD, arc, aroC, proC, argC, nadBE, dapB, trpE, aotJ, phhA, phaF, PA0006, PA0400, PA0440, PA0530, PA1339-PA1342, PA1638, PA2084, PA2108, PA2740, PA3164, PA3271, PA3538, PA3589, PA3871, PA4180, PA4672, PA4774, PA4977, PA5093, PA5522*
Amino acid and Fatty acid metabolism	*gcdH*
Carbohydrate transport and metabolism	*rpe, eno, ppsA, tpiA, pykF, pgm, chiC, PA3430*
Nucleotide transport and metabolism	*cyaA, ndk, guaA, xpt, xdhA, purHLMN, pyrDEFH Q, nrdD,cmk, PA0148, PA0387, PA0439, PA3004, PA3516-PA3517,PA4645*
Amino acid and Nucleotide metabolism	*carAB, prs, purBF, pyrB, PA4758*
Coenzyme transport and metabolism	*cobABLV, folE1E2X, pdxJ, moaA1A2C, thiDl*
Transcription regulators	*rpoD, rpoN, PA0436, PA0512-PA0513, PA0515, PA1201,PA1399, PA1630, PA1859, PA2273, PA2432, PA2885, PA5403*
EPS and LPS synthesis and secretion	*algABCDEFGIJLQRUWXZ, alg8, alg44, kdsA, pelABD, ddlA, pslB, mucBCD, wzt, waaACFG, rfaDE, wbpMWZ, wapR*, *lpxDO2, rmd, rmlC, PA1390, PA3242, PA3256, PA5238, PA5291*
Aerobic and Anaerobic respiration	*azuA, narHIJK1, nasA, nirBCDFJLMNQS, ackA, nosDRZ, rubB napCF, nuoD, aer, ubiA, ercS, norD, etfB, hemE, PA0510, PA0516, PA0918, PA1779, PA3025, PA3491, PA4772, PA5491*
Bacteriophage production	*PA0616-PA0623, PA0627, PA063*
Tricarboxylic acid (TCA) cycle	*fumC1, acnA*
*N*-AHLs and PQS QS-dependent genes and Virulence	*lasIR, rhlI, qscR, rsaL, vfr, pqsR (mvfR), pchR, gacA,pqsBCDEH, antAB, rpoS, xcpTVW, secA, pscT, pcrD, aprA,toxA, katAB, sodBM, lasB, phzD1D2M, phoA, Hxc VUW,PA0682, PA0628, PA0684-PA0685, PA0687, PA4304*
Signal transduction mechanisms	*irlR, pprAB, mifS, bfiRS, PA0431, PA0756, PA0847, PA0930,PA1107, PA1335-PA1336, PA1437-PA1438, PA1782, PA2479-PA2480, PA2524, PA2572, PA2656, PA2766, PA2824, PA3077- PA3078, PA3376, PA3946, PA4197, PA4332, PA4886, PA5487*
Protection and Adaptation	*pcoB, csaA, sspB, surE, trxA, tpx, ahpCF, recA, hslV, grpE, copRS, czcR, phoP, pmrAB, parR, betC, envZ-ompR, lon, asrA, PA0456, PA0961, PA0706, PA2092, PA2521, PA2701, PA2812, PA4218, PA4222-PA4223, PA4775, PA5159, PA5471*
Membrane component and transporters	*pstAB, opdH, exbB1, fiuA, PA0581, PA0751-PA0752, PA0754, PA2042, PA2350, PA2658-PA2659, PA3212, PA3376, PA3671*
Replication, recombination and repair	*gyrA, mutL, uvrA, hepA, recG, mica, ruvB, asp*
Transcription	*rpoACNZ, rhlB, PA2840*
Translation	*infBC, tsf, truB, pnp, miaA, rnd, PA0733, PA1678, PA4673*
Post-translational modification	*dnaJK, clpB, clpXP, clpP2, tig, gcp, dsbG, msrA, PA0473,PA2478, PA3450*
Cell cycle	*ftsHZY, gidA, mreB, cafA, mind*
Hypothetical proteins	*PA0089, PA0193, PA0333, PA0338, PA0429, PA0433, PA0581, PA0754, PA0841, PA0935, PA0939, PA0979, PA0982, PA1016, PA1096, PA1149, PA1167, PA1367, PA1571, PA1814, PA1925, PA2137, PA2189, PA2412, PA2464, PA2506, PA2651, PA2659, PA2689, PA2771, PA2780, PA2829, PA2842, PA2958, PA2971, PA3070, PA3143, PA3216, PA3219, PA3229, PA3273, PA3402, PA3762, PA3772, PA3793, PA3869, PA3962, PA4149, PA4202, PA4298-PA4299, PA4395, PA4399, PA4404-PA4405, PA4510, PA4562,PA4884, PA4962, PA5237, PA5463, PA5492, PA5536*

* *The detailed information is provided in Tables S2, S3*.

**Figure 3:**
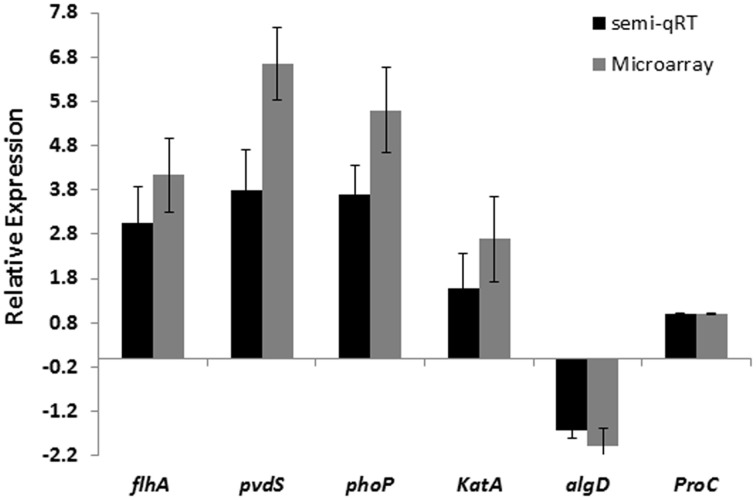
**Comparison of microarray and semi-qRT PCR analyses of 5 selected genes in *P. aeruginosa* biofilm cells exposed to 100 nM CDA and in untreated biofilm cells**. Error bars indicate SD (*n* = 3).

The detected changes in expression were supported by semi-quantitative RT-PCR analysis for *algD*, *katA, flhA, phoP, pvdS* and housekeeping gene, *proC*, from established biofilms after exposure to 100 nM CDA, confirming the validation of the corresponding microarray analysis (Figure 3).

### Genes and PPI networks for DE genes

**Figure 4:**
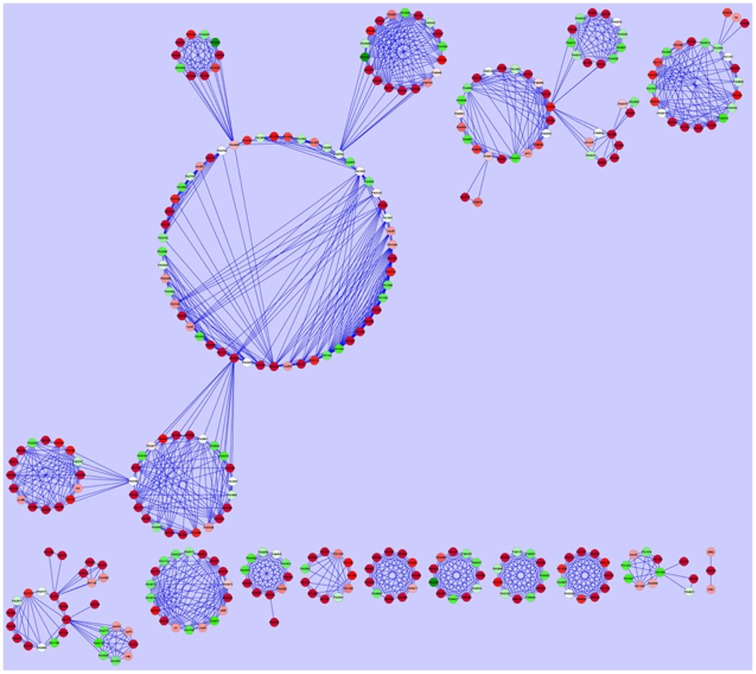
**Protein-protein interaction network construction**. Each gene is represented as a node. Red nodes were up regulated in the presence of CDA, green nodes were down regulated and white ones had no changes in their expression.

Following construction of an interaction network using experimentally proven PPIs for the DE genes with known function, we were able to predict the genes and cellular processes which are linked together and are expressed in the presence of CDA (Barabasi and Oltvai, 2004; Sharan et al., 2007; Dittrich et al., 2008). As it contained many nodes and interactions, we mined the modules in the PPI network to draw the useful information (Figure 4).

**Figure 5:**
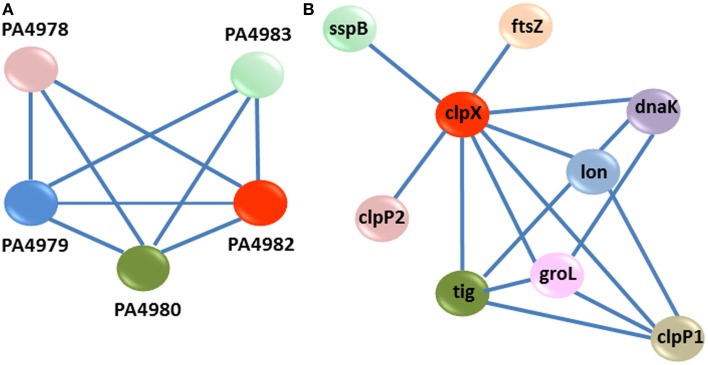
**PPI modules. (A)** Interaction sub-network for synthesis, sensing and response to CDA. Each gene is represented as a node. There are three key genes *PA4980, PA4982*, and *PA4983* in this cluster. *PA4980* encodes a putative enoyl-CoA hydratase/isomerase, required for the synthesis of fatty acid signal. The *PA4982/PA4983* genes pair encodes a two-component system comprising the sensor kinase, *PA4982*, and the response regulator, PA4983, for the perception of signal. **(B)** PPI sub-network for clpX upon exposure to CDA. ClpX regulates the expression of several CDA targeted genes, which belong to a series of cellular processes for instance; adaptation and protection (*sspB*, *lon*, and *groL*), cell cycle (*ftsZ*), and other post-transcriptional modification genes (*clpP1, clpP2, tig*, and *dnaK*).

One of the modules suggested that a cluster of *P. aeruginosa* genes is involved in the synthesis, sensing and responding to CDA (Yang and Lu, 2007). This cluster contains 5 genes (*PA4978, PA4979, PA4980*, *PA4982*, *PA4983*); in which *PA4980* encodes a putative enoyl-CoA hydratase/isomerase that is necessary for synthesis of a fatty acid signal and the *PA4982/PA4983* gene pair encodes a two component system comprising the sensor kinase, *PA4982*, as well as the response regulator, *PA4983*, for the perception of signal (Yang and Lu, 2007) (Figure 5A). While, microarray analysis showed that 8 genes encoding putative enoyl-CoA hydratase/isomerase (*PA3591, PA1021, PA1629, PA2890, PA3426, PA4330, PA1240*, and *PA2841*) were highly up-regulated in our study, known PPI data showed that only PA4980 directly interacts with a sensor kinase and its response regulator (Yang and Lu, 2007). Similarly, microarray analysis disclosed that more than 40 genes encoding sensor kinases were up-regulated in the presence of CDA (Table 1 and Table S2), indicating that there may be more than one sensor for fatty acid signal perception in *P. aeruginosa*. However, known PPI data showed that among all these genes, only PA4983 is connected to a putative enoyl-CoA hydratase/isomerase encoding gene (Yang and Lu, 2007), which is necessary for synthesis of a DSF signal. PPI data also indicated that in addition to *PA4980, PA4982/PA4983* are also interacting with *PA4978* and *PA4979*, encoding an acyl-coA synthetase and a probable acyl-CoA dehydrogenase, respectively (Yang and Lu, 2007) (Figure 5).

Another module predicted that *PA1802*, encoding an ATP- dependent serine protease, ClpX, regulates the expression of several CDA-targeted genes, which belong to a series of cellular processes including; adaptation and protection (*sspB*, *lon*, and *groL*) (Bertani et al., 2007; Breidenstein et al., 2008; de Bruijn and Raaijmakers, 2009), cell cycle (*ftsZ*) (Dajkovic et al., 2008), and other post-transcriptional modification genes (*clpP1, clpP2*, *tig*, and *dnaK*) (Qiu et al., 2008; de Bruijn and Raaijmakers, 2009) (Figure 5B).

### Removal of established biofilms using combined CDA and antibiotics/disinfectants treatments

**Figure 6:**
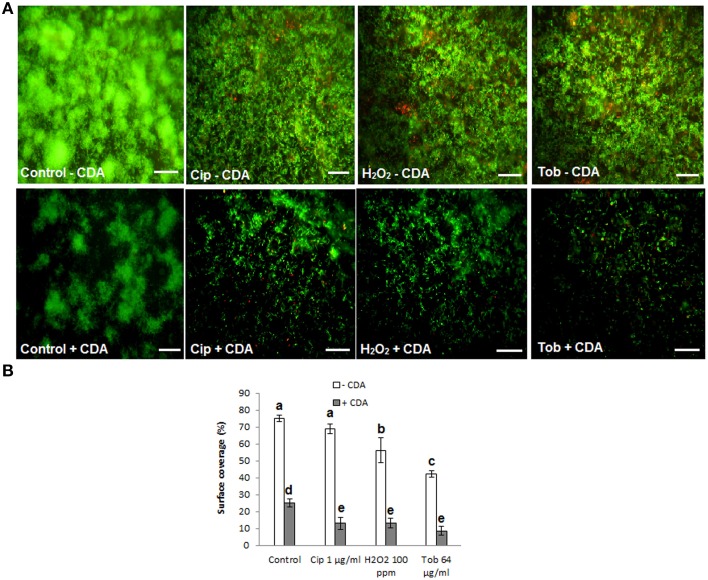
**Removal of established biofilms of *P. aeruginosa*, using CDA combined by antimicrobial treatments**. Following dispersion of biofilms by CDA, cells remaining on the surface were easily killed and removed by various antimicrobial compounds (tobramycin; Tob, H_2_O_2_, ciprofloxacin; Cip). **(A)** LIVE/DEAD staining of biofilms. 48 h-old-biofilms of *P. aeruginosa* were treated with indicated antimicrobials alone or combined with 100 nM CDA for only 1 h. Biofilms were then stained with LIVE/DEAD staining and **(B)** Quantification of percent surface coverage. After LIVE/DEAD staining percent surface coverage was quantified using digital image analysis. Images are top-down views (x-y plane); scale bars: 50 μm. Error bars indicate SD (*n* = 3).

Results from biofilm dispersal bioassays showed that CDA significantly (*P* < 0.05) induces the transition of biofilms to their planktonic phenotypes that are reportedly much more susceptible to antimicrobial agents compared to their biofilm counterparts (Barraud et al., 2006). Moreover, data from microarray analysis revealed that CDA mediates dispersion of biofilms through signaling pathways, including up-regulation of genes involved in motility (e.g., *fliCDFGJMNOQR, flgBCDEGHI, flhAB*), metabolic activity (e.g., *fabABDF1F2GH2IZ, pauA3A5, purHLMN, pyrDEFH, pykF*) and EPS alginate degradative enzyme (*algL*), in addition to down-regulation of genes contributing to attachment (e.g., *cupA3A4B2B5*) and the core gene of EPS alginate synthesis (*algD*) (Sauer et al., 2004; Barraud et al., 2009a). Therefore, we then reasoned that treatment of biofilms using CDA as an inducer of biofilm dispersal, in combination with antibiotics or disinfectants, would be an effective strategy to remove established biofilms. To test this hypothesis, we examined the efficiency of two broadly used antibiotics (tobramycin and ciprofloxacin) as well as hydrogen peroxide (H_2_O_2_) alone or combined with 100 nM CDA on biofilm removal. When 48-h biofilms of *P. aeruginosa* were treated in the absence of CDA, neither the antibiotics nor the disinfectant (H_2_O_2_) were able to exert a significant effect on the percentage of surface area covered by the biofilms (assessed by LIVE/DEAD staining kit) (Figure 6A); however, treatments with a combination of CDA and antibiotic or disinfectant resulted in almost-complete removal of biofilm cells in all cases, as quantified using the digital image analysis method. Besides, we found that following exposure of the biofilms to CDA, the remaining cells on the surface could be easily killed by antimicrobial compounds (quantified using digital image analysis) (Figure 6B).

To further investigate the effect of CDA on the sensitivity of *P. aeruginosa* cells toward antibiotics and disinfectants, we examined the effect of CDA combined treatments on planktonic cells (Rahmani-Badi et al., 2014; Sepehr et al., 2014). We found that compared to use of antibiotics or disinfectants alone, combinational treatments using 100 nM CDA had no additional inhibitory effects on the growth of planktonic cells where the MICs for tobramycin, ciprofloxacin and H_2_O_2_ both in the presence and absence of CDA were 4 μg/ml, 0.25 μg/ml, and 10 ppm, respectively.

## Discussion

A previous work has established that *P. aeruginosa* produces a medium chain fatty acid characterized as CDA (Davies and Marques, 2009). This unsaturated fatty acid acts as an inter-kingdom signal and is capable of inducing biofilm dispersal in several types of microorganisms (Davies and Marques, 2009; Rahmani-Badi et al., 2014; Sepehr et al., 2014). However, a little is known about the mechanism of signal perception and the full range of genes controlled by signaling. Here, we have conducted a microarray gene expression analysis experiment on the *P. aeruginosa* biofilms in the presence and absence of CDA, to determine the scope of genes and biological functions regulated by the signal.

Our findings revealed that CDA signaling plays an important role in regulation of 666 genes (Table 1, Tables S2, S3) encoding proteins and enzymes involved in at least 15 cellular processes, including motility and chemotaxis, attachment, TCA cycle, synthesis and secretion of EPS and LPS, virulence, persistence at different temperatures, iron uptake, aerobic and anaerobic respiration, bacteriophage production and others.

These findings suggest a vital role for CDA-signaling in keeping up the general competence of *P. aeruginosa* in different ecosystems (He et al., 2006). These results were highly consistent with those obtained from genome scale microarray analysis of DSF regulon in *Xcc* that were shown to play a key role in the regulation of almost 200 important genes involved in more than 12 functional groups (He et al., 2006).

CDA-regulated genes are randomly distributed throughout the *P. aeruginosa* genome. This pattern is also similar to genes modulated by DSF in *Xcc* that are randomly scattered in the genome, indicating a conserved role for fatty acid-signaling in pathogenic bacteria (He et al., 2006).

**Figure 7:**
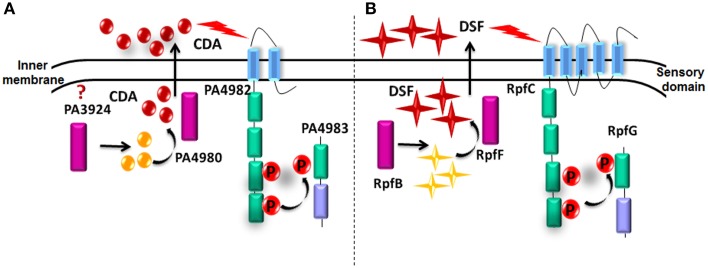
**Comparison of predicted and known proteins involved in CDA and DSF synthesis and perception. (A)** CDA synthesis and perception in *P. aeruginosa* based on known PPIs. **(B)** Demonstrated DSF synthesis and perception in *Xcc*.

A BLAST search using the rpfF/rpfR gene cluster showed that this sensor/signal gene pair is highly conserved in various bacterial pathogens, comprising members of the genera *Xanthomonas*, *Enterobacter*, *Thiobacillus*, *Xylella*, *Serratia*, *Leptospirillum*, *Stenotrophomonas*, *Burkholderia*, *Achromobacter*, *Yersinia*, *Methylobacillus*, *Pantoea*, and *Cronobacter* (Deng et al., 2012). However, it failed to find an *rpf* gene cluster or protein closely related to RpfF in *P. aeruginosa*. Instead, known PPI data suggested that a cluster of 5 genes (*PA4978, PA4979, PA4980*, *PA4982-PA4983*) are involved in the synthesis, sensing and responding to CDA. In this cluster, *PA4982* encodes a sensor kinase (Figure 7). In agreement with these results, Davies and Marques (2009) in their study reported that *P. aeruginosa* has 155 sensor kinases, of which *PA4982* has the highest sequence identity with RpfC (32% identity over 538 amino acids, *E*-value of 2.0 E-72) and is likely involved in perception of CDA. However, the sensory domain of PA4982 is different from that of the DSF sensor in xanthomonads, RpfC (McCarthy et al., 2010). Bioinformatics analysis predicts that RpfC has 5 trans- membrane helices with peri-plasmic and cytoplasmic loops of less than 20 amino acids (McCarthy et al., 2010; Ryan and Dow, 2011) (Figure 7). In contrast, PA4982 is predicted to have 2 trans-membrane helices and a large peri-plasmic loop of 111 amino acids (McCarthy et al., 2010) (Figure 7). The mechanism by which sensor recognizes CDA is yet unknown. Transduction of the DSF signal in xanthomonads comprises modulation of the second messenger cyclic di-GMP levels through the action of RpfG, an HD-GYP domain regulator that has cyclic di-GMP phosphodiesterase activity (McCarthy et al., 2010; Ryan and Dow, 2011). Although there is no information regarding the CDA signal transduction mechanism, we speculate that this may include *PA4983* that encodes a CheY-like receiver domain with winged-helix DNA-binding domain, instead of an RpfG-like regulator (McCarthy et al., 2010; Ryan and Dow, 2011).

In this cluster *PA4982/PA4983* also interacts with *PA4980* encoding a putative enoyl-CoA hydratase/isomerase, which is necessary for synthesis of a DSF signal (Yang and Lu, 2007). In contrast to our results recently Amari et al., have reported that *PA0745* which encodes a putative enoyl-CoA hydratase/isomerase with 30% sequence identity to Bcam0581 (the protein responsible for DSF synthesis in *Burkholderia cenocecepacia)* is involved in production of CDA (2013). Even though our microarray analysis also indicated an up regulation in *PA0745* in the presence of CDA, known PPI data did not show any interaction between PA4982/PA4983 and PA0745 (Yang and Lu, 2007). While microarray analysis showed that *PA3924*, which encodes a probable medium-chain acyl-CoA ligase, was up regulated in the presence of CDA, known PPI data did not show any interaction with *PA4982/PA4983* (Yang and Lu, 2007). Based on these results, we hypothesize that for sensing and responding to CDA, the sensor kinase PA4982 interacts with PA4980 to modulate CDA signal production and after reaching a threshold concentration at extracellular environment, CDA signal is transduced through PA4982 sensor to PA4983 response regulator via a phosphorelay mechanism (He et al., 2007). Further experiments such as mutational studies are needed to support proposed functions for this cluster of genes.

PPI data also predicted that ClpX, an ATP-dependent serine protease, is involved in a series of CDA-regulated biological functions. This finding is in agreement with previous works demonstrating that intracellular proteases such as ClpXP are involved in several biological functions in some pathogenic bacteria, including *E. coli* (Iyoda and Watanabe, 2005), Salmonella (Tomoyasu et al., 2002) and Bacillus (Msadek et al., 1998). Moreover, in a previous investigation it was also shown that ClpXP regulates the synthesis of alginate in *P. aeruginosa* (Qiu et al., 2008).

Since *P. aeruginosa* also uses *N*-AHLs as intracellular signals to regulate gene expression through the two LuxR families, it raises the issue as to what extent the regulatory influences of the two classes of signaling pathway overlap. Comparison of the CDA gene regulation scope described here, with those genes regulated by *lasIR* and *rhlIR* (Wagner et al., 2003) showed that out of 550 up regulated genes in presence of CDA, only 23 genes were responsive to *N*-AHLs. While only one gene from the down regulated gene list in the presence of CDA was responsive to *N*-AHLs. This indicates that little shared mechanism exist between these two signaling pathways.

The discovery of a signaling molecule responsible for biofilm dispersion with inter-kingdom activity has significant implications for the exogenous induction of the transition of microbial biofilms to their planktonic state (Barraud et al., 2009b; Davies and Marques, 2009). In fact, the unusual resistance of microbial biofilms to treatment with antimicrobial agents and the persistence and chronic nature of biofilm-associated infections could potentially be reversed if, in treatment, microbial biofilms could be forced to transition to their planktonic phenotypes (Barraud et al., 2009b; Davies and Marques, 2009). Therefore, the application of a dispersion inducer prior to, or in combination with, treatment by conventional antimicrobial agents would be able to provide a novel mechanism for enhancing the activity of these treatments through the disruption of existing biofilms (Barraud et al., 2009b; Davies and Marques, 2009). Thus, in this study we tested three common antimicrobial agents alone or in combination with 100 nM CDA against *P. aeruginosa* biofilms. Our results showed that combinational treatments using both CDA and antibiotics or H_2_O_2_ could result in almost-complete removal of the biofilms where the minimum bactericidal concentration (MIC) for tobramycin that was reportedly over 400 μg/ml for *P. aeruginosa* biofilms grown on an abiotic surface (Moreau-Marquis et al., 2009), decreased to only 64 μg/ml tobramycin (when combined with CDA). Similar results were observed in the case of ciprofloxacin and H_2_O_2_ as well.

Even though several previous studies have shown that some free fatty acids have antimicrobial activities (Desbois et al., 2008; Desbois and Smith, 2010) and play a significant role in keeping up the microbial flora of the skin (Takigawa et al., 2005; Kenny et al., 2009), we confirmed that CDA does not inhibit bacterial growth at low concentrations (nano-molar ranges). These results were highly in agreement with data obtained from a previous study (Jennings et al., 2012), indicating that CDA only can inhibit bacterial growth at high concentrations (micro-molar to milli-molar). This lack of growth inhibition at nano-molar concentrations was not surprising since *P. aeruginosa* produces this fatty acid and uses it as a signaling molecule (Davies and Marques, 2009; Rahmani-Badi et al., 2014; Sepehr et al., 2014).

## Conclusions

Since CDA induces the transition from a biofilm to a planktonic phenotype via a signaling mechanism instead of a toxic effect (Barraud et al., 2009b; Davies and Marques, 2009; Rahmani-Badi et al., 2014), CDA-based biofilm control strategies are not expected to result in selection of resistant strains, as seen in antibiotics treatment methods (Rahmani-Badi et al., 2014). Moreover, these strategies would involve the use of very low concentrations of CDA, nano-molar ranges that is expected to be safe to humans and to the ecosystem. In addition, in a previous study Jennings et al. (2012) have shown that CDA has no cytotoxic or stimulatory effect on human cells, even at higher concentrations (up to 250 μg/ml). In their study it was also suggested that local delivery of CDA to injured tissues could potentially prevent the clinically biofilm formation (Jennings et al., 2012). Overall, anti-biofilm properties of CDA, as well as the capability of delivering CDA from a biocompatible local delivery system indicates its great potential for clinical investigation and uses (Jennings et al., 2012).

These findings have laid down valuable frameworks for developing promising strategies against biofilm-associated infections.

### Conflict of interest statement

The authors declare that the research was conducted in the absence of any commercial or financial relationships that could be construed as a potential conflict of interest.
