# Supplementary material for: Dissection of the cis-2-decenoic acid signaling network in Pseudomonas aeruginosa using microarray technique
Source: Front Microbiol. 2015 Apr 28;6:383. doi: 10.3389/fmicb.2015.00383 (PMC4412052; doi:10.3389/fmicb.2015.00383)
Supplement: Supplementary file 1 [file Table1.DOCX]

**Supplemental Table1.** Primers used in this study

| Primer | Sequence (5’-3’) |
| --- | --- |
| *algD*-F | ggctatgtcggtgcagtatg |
| *algD*-R | cttcttgaagtcggtggtgc |
| *katA*-F | tcaagttccccgatctcaac |
| *katA*-F | gaagctgaaggtatggctgc |
| *flhA*-F | tgccgcatgtttccttcatc |
| *flhA*-R | aacccagttccttgacctcc |
| *phoP*-F | actacgtggtcaagccgttc |
| *phoP*-R | gaggatgcggtattcgtagg |
| *pvdS-*F | accgtacgatcctggtgaag |
| *pvdS*-R | gtagtgatcgatggccaggt |
| *proC*-F | caggccgggcagttgctgtc |
| *proC*-R | ggtcaggcgcgaggctgtct |
