# Supplementary material for: Dissection of the cis-2-decenoic acid signaling network in Pseudomonas aeruginosa using microarray technique
Source: Front Microbiol. 2015 Apr 28;6:383. doi: 10.3389/fmicb.2015.00383 (PMC4412052; doi:10.3389/fmicb.2015.00383)
Supplement: Supplementary file 2 [file Table2.DOCX]

**Supplemental Table 2.** Genes up-regulated by CDA in *P. aeruginosa* PAO1 determined through microarray analysis. The ratio of gene expression in untreated biofilm cells to that of CDA treated biofilm cells (fold up-regulation) is displayed for genes that exhibited changes >1-fold. Ratios are the average of three independent experiments.

| Fold changes | Function or class | Gene | ORF or operon |
| --- | --- | --- | --- |
| 6.39 | chemotaxis protein MotA | *motA* | PA4954 |
| 4.18 | chemotaxis protein MotB | *motB* | PA4953 |
| 3.31 | MotC | *motC* | PA1460 |
| 2.90 | threonyl-tRNA synthetase | *thrS* | PA2744 |
| 2.41 | Transketolase | *tktA* | PA0548 |
| 14.03 | probable chemotaxis transducer |  | PA4844 |
| 2.18 | probable enoyl-CoA hydratase /isomerase |  | PA3591 |
| 5.57 | CDP-diacylglycerol-glycerol-3-phosphate | *pgsA* | PA2584 |
| 3.96 | probable methylesterase |  | PA0173 |
| 17.71 | Cluster II che genes for optimal chemotaxic response |  | PA0174 |
| 4.65 | probable chemotaxis protein methyltransferase, Cluster II che genes for optimal chemotaxic response |  | PA0175 |
| 2.68 | aerotaxis transducer Aer2, Cluster II che genes for optimal chemotaxic response | *aer2* | PA0176 |
| 2.40 | probable purine-binding chemotaxis protein, Cluster II che genes for optimal chemotaxic response |  | PA0177 |
| 1.28 | probable purine-binding chemotaxis protein, Cluster II che genes for optimal chemotaxic response |  | PA0178 |
| 1.09 | probable two-component response regulator, Cluster II che genes to optimal chemotaxic response |  | PA0179 |
| 1.03 | probable chemotaxis transducer |  | PA0180 |
| 1.22 | phosphomannose isomerase / guanosine | *algA* | PA3551 |
| 4.60 | two-component response regulator AlgB | *algB* | PA5483 |
| 4.39 | Alginate production outer membrane protein AlgE | *algE* | PA3544 |
| 4.93 | alginate o-acetyltransferase AlgF | *algF* | PA3550 |
| 30.84 | alginate o-acetyltransferase AlgI | *algI* | PA3548 |
| 1.18 | alginate o-acetyltransferase AlgJ | *algJ* | PA3549 |
| 3.29 | poly(beta-d-mannuronate) lyase precursor AlgL | *algL* | PA3547 |
| 1.66 | Alginate regulatory protein AlgQ | *algQ* | PA5255 |
| 3.50 | sigma factor AlgU | *algU* | PA0762 |
| 1.44 | AlgW protein | *algW* | PA4446 |
| 3.95 | alginate biosynthesis protein AlgX | *algX* | PA3546 |
| 11.83 | alginate biosynthesis protein AlgZ/FimS | *algZ* | PA5262 |
| 4.76 | alginate biosynthesis protein Alg8 | *alg8* | PA3541 |
| 3.05 | alginate biosynthesis protein Alg44 | *alg44* | PA3542 |
| 3.60 | probable very-long-chain acyl-CoA synthetase |  | PA2893 |
| 4.14 | 30S ribosomal protein S2 | *rpsB* | PA3656 |
| 7.10 | 30S ribosomal protein S7 | *rpsG* | PA4267 |
| 15.69 | 30S ribosomal protein S9 | *rpsI* | PA4432 |
| 5.52 | 30S ribosomal protein S12 | *rpsL* | PA4268 |
| 1.47 | 30S ribosomal protein S16 | *rpsP* | PA3745 |
| 14.84 | flagellar basal-body rod protein FlgB | *flgB* | PA1077 |
| 3.40 | flagellar basal-body rod protein FlgC | *flgC* | PA1078 |
| 12.59 | flagellar basal-body rod modification protein | *flgD* | PA1079 |
| 1.83 | flagellar hook protein FlgE | *flgE* | PA1080 |
| 2.13 | flagellar basal-body rod protein FlgG | *flgG* | PA1082 |
| 14.59 | flagellar L-ring protein precursor FlgH | *flgH* | PA1083 |
| 7.94 | flagellar P-ring protein precursor FlgI | *flgI* | PA1084 |
| 19.25 | probable bacteriophage protein |  | PA0617 |
| 4.16 | probable bacteriophage protein |  | PA0618 |
| 2.29 | probable bacteriophage protein |  | PA0619 |
| 9.00 | probable bacteriophage protein |  | PA0620 |
| 2.33 | probable bacteriophage protein |  | PA0621 |
| 4.58 | probable bacteriophage protein |  | PA0622 |
| 2.67 | probable bacteriophage protein |  | PA0623 |
| 6.45 | conserved hypothetical protein, phage protein |  | PA0627 |
| 5.05 | general secretion pathway protein G | *xcpT* | PA3101 |
| 2.33 | general secretion pathway protein I | *xcpV* | PA3099 |
| 2.33 | general secretion pathway protein J | *xcpW* | PA3098 |
| 2.81 | phenazine biosynthesis protein PhzD | *phzD1* | PA4213 |
| 1.60 | phenazine biosynthesis protein PhzD | *phzD2* | PA1902 |
| 3.69 | probable phenazine-specific methyltransferase | *phzM* | PA4209 |
| 15.86 | probable enoyl-CoA hydratase /isomerase |  | PA1021 |
| 1.13 | NADH dehydrogenase I chain C,D | *nuoD* | PA2639 |
| 2.51 | DNA gyrase subunit A | *gyrA* | PA3168 |
| 27.49 | pyruvate kinase I | *pykF* | PA1498 |
| 6.62 | glutaryl-CoA dehydrogenase | *gcdH* | PA0447 |
| 5.90 | aspartate carbamoyltransferase | *pyrB* | PA0402 |
| 8.85 | Dihydroorotase | *pyrC* | PA3527 |
| 4.86 | dihydroorotate dehydrogenase | *pyrD* | PA3050 |
| 6.91 | orotate phosphoribosyl transferase | *pyrE* | PA5331 |
| 3.61 | orotidine 5'-phosphate decarboxylas | *pyrF* | PA2876 |
| 3.50 | uridylate kinase | *pyrH* | PA3654 |
| 7.52 | probable two-component sensor |  | PA3946 |
| 2.70 | probable transporter |  | PA4218 |
| 6.76 | probable type II secretion system protein |  | PA0684 |
| 5.18 | transcriptional regulator FleQ | *fleQ* | PA1097 |
| 4.96 | two-component sensor | *fleS* | PA1098 |
| 12.20 | flagellar synthesis regulator FleN | *fleN* | PA1454 |
| 3.48 | flagellin type B | *fliC* | PA1092 |
| 5.18 | flagellar capping protein FliD | *fliD* | PA1094 |
| 5.87 | Flagella M-ring outer membrane protein | *fliF* | PA1101 |
| 3.22 | flagellar motor switch protein FliG | *fliG* | PA1102 |
| 1.72 | flagellar protein FliJ | *fliJ* | PA1105 |
| 2.28 | flagellar motor switch protein FliM | *fliM* | PA1443 |
| 1.03 | flagellar motor switch protein FliN | *fliN* | PA1444 |
| 5.82 | flagellar protein FliO | *fliO* | PA1445 |
| 28.52 | flagellar biosynthetic protein FliQ | *fliQ* | PA1447 |
| 4.13 | flagellar biosynthetic protein FliR | *fliR* | PA1448 |
| 7.02 | probable two-component response regulator |  | PA0756 |
| 1.57 | assimilatory nitrate reductase |  | PA1779 |
| 2.77 | DnaJ protein | *dnaJ* | PA4760 |
| 7.40 | DnaK protein | *dnaK* | PA4761 |
| 4.38 | L-ornithine N5-oxygenase | *pvdA* | PA2386 |
|  | pyoverdine synthetase D | *pvdD* | PA2399 |
| 16.43 | pyoverdine biosynthesis protein PvdE | *pvdE* | PA2397 |
| 4.83 | pyoverdine synthetase F | *pvdF* | PA2396 |
| 9.32 | PvdG | *pvdG* | PA2425 |
| 10.85 | PvdJ | *pvdJ* | PA2400 |
| 2.02 | PvdL | *pvdL* | PA2424 |
| 11.92 | PvdN | *pvdN* | PA2394 |
| 14.40 | PvdO | *pvdO* | PA2395 |
| 15.48 | PvdP | *pvdP* | PA2392 |
| 6.65 | sigma factor PvdS | *pvdS* | PA2426 |
| 4.13 | flagellar biosynthesis protein FlhA | *flhA* | PA1448 |
| 7.80 | flagellar biosynthetic protein FlhB | *flhB* | PA1449 |
| 1.05 | heat shock protein HscA | *hscA* | PA3810 |
| 1.24 | Lon protease | *lon* | PA1803 |
| 1.55 | aconitate hydratase 1 | *acnA* | PA1562 |
| 1.44 | ribonuclease III | *rnc* | PA0770 |
| 2.32 | respiratory nitrate reductase beta chain | *narH* | PA3874 |
| 5.93 | respiratory nitrate reductase gamma chain | *narI* | PA3872 |
| 14.72 | respiratory nitrate reductase delta chain | *narJ* | PA3873 |
| 2.49 | nitrite extrusion protein 1 | *nark1* | PA3877 |
| 1.01 | nitrite extrusion protein 2 | *narK2* | PA3876 |
| 1.70 | probable biotin-dependent carboxylase |  | PA2888 |
| 3.93 | urease gamma subunit | *ureA* | PA4865 |
| 1.11 | two-component sensor EnvZ | *envZ* | PA5199 |
| 3.86 | two-component response regulator OmpR | *ompR* | PA5200 |
| 6.61 | probable two-component sensor |  | PA2524 |
| 3.59 | probable two-component sensor | *parR* | PA1798 |
| 2.14 | carbamoyl-phosphate synthase small chain |  | PA4758 |
| 2.37 | probable phosphomannose isomerase/GDP-mannose | *pslB* | PA2232 |
| 1.37 | two-component response regulator | *irlR* | PA4885 |
| 1.21 | probable enoyl-CoA hydratase /isomerase |  | PA1629 |
| 1.78 | probable short-chain dehydrogenase |  | PA2887 |
| 2.09 | probable chemotaxis transducer |  | PA5072 |
| 2.50 | transcriptional regulator Vfr | *vfr* | PA0652 |
| 1.28 | two-component response regulator PfeR | *pfeR* | PA2686 |
| 5.28 | two-component sensor PfeS | *pfeS* | PA2687 |
| 1.61 | assimilatory nitrite reductase large subunit | *nirB* | PA1781 |
| 16.92 | probable c-type cytochrome precursor | *nirC* | PA0517 |
| 1.88 | assimilatory nitrite reductase small subunit | *nirD* | PA1780 |
| 5.16 | heme d1 biosynthesis protein NirF | *nirF* | PA0516 |
| 8.31 | heme d1 biosynthesis protein NirJ | *nirJ* | PA0511 |
| 9.12 | cytochrome c-551 precursor | *nirM* | PA0518 |
| 18.35 | probable c-type cytochrome | *nirN* | PA0509 |
| 27.45 | nitrite reductase precursor | *nirS* | PA0519 |
| 5.62 | probable dinitrification protein NorD | *norD* | PA0525 |
| 10.20 | probable chemotaxis transducer | *wspA* | PA3708 |
| 3.17 | hypothetical protein | *wspB* | PA3707 |
| 1.39 | probable protein methyl transferase | *wspC* | PA3706 |
| 1.45 | probable chemotaxis sensor/effector fusion | *wspE* | PA3704 |
| 5.03 | probable methylesterase | *wspF* | PA3703 |
| 4.41 | probable two-component response regulator | *wspR* | PA3702 |
| 9.85 | probable chemotaxis transducer |  | PA1646 |
| 18.04 | GroEL protein | *groEL* | PA4385 |
| 8.22 | elastase LasB | *lasB* | PA3724 |
| 2.46 | transcriptional regulator LasR | *lasR* | PA1430 |
| 21.40 | autoinducer synthesis protein LasI | *lasI* | PA1432 |
| 7.08 | nucleotide sugar epimerase /dehydratase WbpM | *wbpM* | PA3141 |
| 1.55 | phosphomannose isomerase/GDP-mannose WbpW | *wbpW* | PA5452 |
| 1.94 | glycosyltransferase WbpX | *wbpX* | PA5449 |
| 6.20 | glycosyltransferase WbpY | *wbpY* | PA5448 |
| 6.50 | glycosyltransferase WbpZ | *wbpZ* | PA5447 |
| 1.99 | folylpolyglutamate synthetase | *folC* | PA3111 |
| 17.86 | GTP cyclohydrolase I precursor | *folE1* | PA3438 |
| 4.21 | GTP cyclohydrolase I precursor | *folE2* | PA1674 |
| 2.89 | S-adenosyl-L-homocysteine hydrolase | *sahH* | PA0432 |
| 2.88 | glyceraldehyde 3-phosphate dehydrogenase | *gapA* | PA3195 |
| 3.21 | aerotaxis receptor Aer | *aer* | PA1561 |
| 388.36 | probable two-component response regulator | *pprB* | PA4296 |
| 4.27 | probable two-component response regulator |  | PA4983 |
| 4.90 | regulatory protein RsaL | *rsaL* | PA1431 |
| 6.40 | glutamine synthetase | *glnA* | PA5119 |
| 1.82 | probable enoyl-CoA hydratase /isomerase |  | PA2890 |
| 2.36 | probable chemotaxis transducer |  | PA2561 |
| 9.06 | ATP-dependent Clp protease proteolytic subunit | *clpP* | PA1801 |
| 7.24 | ATP-dependent Clp protease ATP-binding subunit | *clpX* | PA1802 |
| 5.55 | probable Clp-family ATP-dependent protease | *ClpP2* | PA3326 |
| 1.59 | ClpB protein | *clpB* | PA4542 |
| 2.04 | Homologous to beta-keto-acyl-acyl-carrier | *pqsB* | PA0997 |
| 1.69 | Homologous to beta-keto-acyl-acyl-carrier | *pqsC* | PA0998 |
| 1.10 | 3-oxoacyl-[acyl-carrier-protein] synthase III | *pqsD* | PA0999 |
| 6.96 | Quinolone signal response protein | *pqsE* | PA1000 |
| 1.80 | cell division protein FtsZ | *ftsZ* | PA4407 |
| 2.33 | probable oxidoreductase |  | PA0440 |
| 1.19 | probable medium-chain acyl-CoA ligase |  | PA3924 |
| 3.40 | cytochrome c-type protein NapC | *napC* | PA1172 |
| 6.64 | ferredoxin protein NapF | *napF* | PA1176 |
| 3.34 | trigger factor | *tig* | PA1800 |
| 1.95 | DNA mismatch repair protein MutL | *mutL* | PA4946 |
| 7.51 | guanylate kinase | *gmk* | PA5336 |
| 3.09 | probable oxidoreductase |  | PA0439 |
| 135.80 | probable acyl-CoA dehydrogenase |  | PA1022 |
| 1.34 | probable chemotaxis transducer |  | PA2573 |
| 2.92 | probable two-component response regulator |  | PA2809 |
| 2.04 | two-component response regulator CheY | *cheY* | PA1456 |
| 1.14 | chemotaxis protein CheZ | *cheZ* | PA1457 |
| 2.20 | heat shock protein HslV | *hslV* | PA5053 |
| 5.38 | thioredoxin | *trxA* | PA5240 |
| 28.00 | phenylalanyl-tRNA synthetase, alpha-subunit |  | PA2740 |
| 5.19 | uroporphyrin-III C-methyl transferase | *cobA* | PA1778 |
| 986.02 | -2-dehydro-3-deoxy phosphorroctonate aldolase | *kdsA* | PA3636 |
| 2.21 | triosephosphate isomerase | *tpiA* | PA4748 |
| 5.85 | probable acyl-CoA dehydro genase |  | PA0508 |
| 2.76 | probable two-component sensor |  | PA4982 |
| 2.36 | azurin precursor | *azu* | PA4922 |
| 3.48 | probable two-component sensor |  | PA5512 |
| 3.09 | ADP-L-glycero-D-mannoheptose 6-epimerase | *rfaD* | PA3337 |
| 6.07 | LPS biosynthesis protein RfaE | *rfaE* | PA4996 |
| 1.46 | probable two-component response regulator |  | PA2523 |
| 17.85 | xanthine phospho ribosyl transferase | *xpt* | PA5298 |
| 2.41 | heat shock protein GrpE | *grpE* | PA4762 |
| 4.62 | glycerol-3-phosphate dehydrogenase | *glpD* | PA3584 |
| 4.48 | glycerol kinase | *glpK* | PA3582 |
| 1.37 | DNA-directed RNA polymerase alpha chain | *rpoA* | PA4238 |
| 1.06 | DNA-directed RNA polymerase beta chain | *rpoB* | PA4270 |
| 2.43 | DNA-directed RNA polymerase beta* chain | *rpoC* | PA4269 |
| 6.97 | sigma factor RpoD | *rpoD* | PA0576 |
| 1.31 | RNA polymerase sigma-54 factor | *rpoN* | PA4462 |
| 4.91 | sigma factor RpoS | *rpoS* | PA3622 |
| 33.06 | RNA polymerase omega subunit | *rpoZ* | PA5337 |
| 15.26 | stringent starvation protein B | *sspB* | PA4427 |
| 3.42 | probable antioxidant protein |  | PA3450 |
| 8.54 | probable transcriptional regulator |  | PA0515 |
| 5.60 | two-component response regulator PhoP | *phoP* | PA1179 |
| 1.88 | probable chemotaxis transducer |  | PA4520 |
| 2.25 | autoinducer synthesis protein RhlI | *rhlI* | PA3476 |
| 1.98 | -3-deoxy-D-manno octulosonic-acid (KDO) | *waaA* | PA4988 |
| 1.46 | heptosyltransferase I | *waaC* | PA5011 |
| 35.03 | heptosyltransferase II | *waaF* | PA5012 |
| 3.47 | UDP-glucose:(heptosyl) LPS alpha | *waaG* | PA5010 |
| 4.40 | probable acetate kinase | *ackA* | PA0836 |
| 2.45 | probable chemotaxis transducer |  | PA2652 |
| 1.13 | cell division protein FtsH | *ftsH* | PA4751 |
| 8.38 | probable 3-hydroxyacyl-CoA dehydrogenase |  | PA1628 |
| 1.89 | pyochelin biosynthetic protein PchC | *pchC* | PA4229 |
| 18.25 | pyochelin biosynthesis protein PchD | *pchD* | PA4228 |
| 3.12 | pyochelin biosynthetic protein PchG | *pchG* | PA4224 |
| 3.08 | transcriptional regulator PchR | *pchR* | PA4227 |
| 5.97 | excinuclease ABC subunit A | *uvrA* | PA4234 |
| 1.71 | probable ATP-binding component of ABC |  | PA4222 |
| 4.21 | queuine tRNA-ribosyltransferase | *tgt* | PA3823 |
| 2.92 | NosD protein | *nosD* | PA3393 |
| 4.65 | nitrous-oxide reductase precursor | *nosZ* | PA3392 |
| 5.60 | probable two-component response regulator | *bfiR* | PA4196 |
| 7.17 | probable two-component sensor | *bfiS* | PA4197 |
| 1.22 | ATP-dependent RNA helicase RhlB | *rhlB* | PA3861 |
| 14.65 | probable serine/threonine-protein kinase |  | PA1782 |
| 9.95 | ABC subunit of A-band LPS efflux transporter | *wzt* | PA5450 |
| 23.27 | RecA protein | *recA* | PA3617 |
| 2.91 | probable enoyl CoA-hydratase /isomerase |  | PA3426 |
| 3.13 | aspartate kinase alpha and beta chain | *lysC* | PA0904 |
| 5.24 | pyridoxal phosphate biosynthetic protein PdxJ | *pdxJ* | PA0773 |
| 3.76 | probable lauroyl acyltransferase |  | PA3242 |
| 2.37 | alkyl hydroperoxide reductase subunit C | *ahpC* | PA0139 |
| 14.64 | alkyl hydroperoxide reductase subunit F | *ahpF* | PA0140 |
| 1.13 | probable short-chain dehydrogenase |  | PA5524 |
| 8.92 | probable chemotaxis transducer |  | PA2654 |
| 7.78 | imidazoleglycerol-phosphate dehydratase | *hisB* | PA5143 |
| 10.50 | D-amino acid dehydrogenase, small subunit | *dadA* | PA5304 |
| 2.30 | translocation protein in type III secretion | *pscT* | PA1691 |
| 275.75 | superoxide dismutase | *sodB* | PA4366 |
| 3.48 | superoxide dismutase | *sodM* | PA4468 |
| 1.58 | translation initiation factor IF-2 | *infB* | PA4744 |
| 2.77 | translation initiation factor IF-3 | *infC* | PA2743 |
| 1.68 | probable purine/pyrimidine phosphoribosyl |  | PA4645 |
| 6.93 | probable transcriptional regulator |  | PA1630 |
| 5.89 | probable transcriptional regulator |  | PA2273 |
| 8.13 | glutamyl-tRNA synthetase | *gltX* | PA3134 |
| 1.03 | probable chemotaxis transducer |  | PA0180 |
| 6.87 | probable type II secretion system protein |  | PA0685 |
| 7.17 | probable enoyl-CoA hydratase/isomerase |  | PA4330 |
| 5.42 | probable sigma-70 factor, ECF subfamily |  | PA2468 |
| 6.77 | probable chemotaxis transducer |  | PA4290 |
| 2.03 | probable short-chain dehydrogenase |  | PA1827 |
| 3.14 | RNA helicase HepA | *hepA* | PA3308 |
| 3.06 | anthranilate dioxygenase large subunit | *antA* | PA2512 |
| 27.67 | anthranilate dioxygenase small subunit | *antB* | PA2513 |
| 36.12 | probable two-component response regulator |  | PA2479 |
| 5.85 | O-sialoglycoprotein endopeptidase | *gcp* | PA0580 |
| 1.68 | probable two-component sensor |  | PA1438 |
| 3.31 | probable thiol:disulfide interchange protein |  | PA2478 |
| 1.70 | probable FAD-dependent glycerol-3-phosphate |  | PA3025 |
| 1.99 | adenylosuccinate lyase | *purB* | PA2629 |
| 12.01 | phosphoribosylamine-glycine ligase | *purD* | PA4855 |
| 33.96 | amidophosphoribosyltransferase | *purF* | PA3108 |
| 6.99 | phosphoribosylformylglycinamidine synthase | *purL* | PA3763 |
| 8.58 | phosphoribosylaminoimidazole synthetase | *purM* | PA0945 |
| 3.24 | phosphoribosylaminoimidazole synthetase | *purN* | PA0944 |
| 2.17 | probable acyl-CoA dehydrogenase |  | PA1187 |
| 2.97 | probable ATP-dependent protease |  | PA0779 |
| 1.65 | glycine cleavage system protein T2 | *gcvT2* | PA2442 |
| 1.24 | beta subunit of geranoyl-CoA carboxylase, GnyB | *gnyB* | PA2014 |
| 1.26 | anthranilate synthetase component | *trpE* | PA0609 |
| 6.09 | probable two-component response regulator |  | PA3947 |
| 2.67 | type 4 prepilin peptidase PilD | *pilD* | PA4528 |
| 7.80 | type 4 fimbrial biogenesis protein PilE | *pilE* | PA4556 |
| 8.72 | type 4 fimbrial biogenesis protein PilM | *pilM* | PA5044 |
| 2.30 | type 4 fimbrial biogenesis protein PilP | *pilP* | PA5041 |
| 3.10 | two-component response regulator PilR | *pilR* | PA4547 |
| 10.53 | two-component sensor PilS | *pilS* | PA4546 |
| 3.27 | twitching motility protein PilU | *pilU* | PA0396 |
| 3.16 | type 4 fimbrial biogenesis protein PilZ | *pilZ* | PA2960 |
| 2.33 | PmrA: two-component regulator system response | *pmrA* | PA4776 |
| 15.77 | PmrB: two-component regulator system signal | *pmrB* | PA4777 |
| 10.88 | ATP-binding component of ABC phosphate | *pstB* | PA5366 |
| 5.62 | glycyl-tRNA synthetase alpha chain | *glyQ* | PA0009 |
| 18.81 | oxidoreductase Rmd | *rmd* | PA5454 |
| 31.90 | probable two-component response regulator |  | PA3077 |
| 4.28 | GTP cyclohydrolase II / 3,4-dihydroxy-2-butanone | *ribB* | PA4054 |
| 3.98 | probable two-component sensor |  | PA1458 |
| 13.82 | probable ATP-binding component of ABC |  | PA1339 |
| 4.83 | glucose-inhibited division protein A | *gidA* | PA5565 |
| 2.35 | rod shape-determining protein MreB | *mreB* | PA4481 |
| 9.76 | alkaline phosphatase | *phoA* | PA3296 |
| 7.89 | probable two-component sensor |  | PA4293 |
| 2.19 | Pvds-regulated endoprotease, lysyl class | *prpL* | PA4175 |
| 16.76 | probable glycosyl transferase |  | PA0705 |
| 11.03 | probable lyase |  | PA3516 |
| 3.90 | nucleoside diphosphate kinase | *ndk* | PA3807 |
| 6.15 | beta-hydroxydecanoyl-ACP dehydrase | *fabA* | PA1610 |
| 3.09 | beta-ketoacyl-ACP synthase I | *fabB* | PA1609 |
| 2.85 | malonyl-CoA-[acyl-carrier-protein] transacylase | *fabD* | PA2968 |
| 3.64 | beta-ketoacyl-acyl carrier protein synthase II | *fabF1* | PA2965 |
| 2.87 | 3-oxoacyl-acyl carrier protein synthase II | *fabF2* | PA1373 |
| 30.47 | 3-oxoacyl-[acyl-carrier-protein] reductase | *fabG* | PA2967 |
| 4.80 | 3-oxoacyl-[acyl-carrier-protein] synthase III | *fabH2* | PA3333 |
| 7.34 | NADH-dependent enoyl-ACP reductase | *fabI* | PA1806 |
| 5.26 | (3R)-hydroxymyristoyl-[acyl carrier protein] | *fabZ* | PA3645 |
| 2.28 | probable two-component sensor |  | PA1992 |
| 1.19 | glutamate dehydrogenase | *gdhA* | PA4588 |
| 8.16 | methionine adenosyltransferase | *metK* | PA0546 |
| 2.05 | ATP-dependent DNA helicase RecG | *recG* | PA5345 |
| 2.12 | dihydrodipicolinate reductase | *dapB* | PA4759 |
| 1.25 | NH3-dependent NAD synthetase | *nadE* | PA4920 |
| 1.39 | hypothetical protein | *pelD* | PA3061 |
| 1.53 | probable type II secretion system protein |  | PA0687 |
| 3.40 | cytochrome c-type protein NapC |  | PA1172 |
| 6.25 | N-acetyl-gamma-glutamyl-phosphate reductase | *argC* | PA0662 |
| 6.94 | probable fatty acid desaturase |  | PA0286 |
| 1.75 | ketol-acid reductoisomerase | *ilvC* | PA4694 |
| 3.41 | dihydroxy-acid dehydratase | *ilvD* | PA0353 |
| 3.42 | probable acyl-CoA dehydrogenase |  | PA2815 |
| 3.96 | probable acyl-CoA dehydrogenase |  | PA3593 |
| 2.39 | probable carbohydrate kinase |  | PA3579 |
| 1.32 | probable acyl-CoA dehydrogenase |  | PA1631 |
| 9.01 | probable transcriptional regulator |  | PA0513 |
| 12.50 | probable binding protein component of ABC |  | PA1342 |
| 5.42 | probable acyl carrier protein |  | PA1869 |
| 1.41 | lysophosphatidic acid acyltransferase, LptA | *lptA* | PA0005 |
| 4.76 | probable short-chain dehydrogenase |  | PA0182 |
| 14.52 | elongation factor Ts | *tsf* | PA3655 |
| 9.53 | probable cytochrome c oxidase subunit |  | PA0521 |
| 2.78 | probable permease of ABC transporter |  | PA1341 |
| 2.34 | probable acyl-CoA dehydrogenase |  | PA2550 |
| 1.83 | valyl-tRNA synthetase |  | PA3834 |
| 4.85 | probable uroporphyrin-III c-methyltransferase |  | PA0510 |
| 1.42 | probable glutamine synthetase |  | PA5522 |
| 2.51 | probable acyl-CoA dehydrogenase |  | PA4995 |
| 6.37 | two-component sensor PhoR | *phoR* | PA5361 |
| 1.39 | phosphate uptake regulatory protein PhoU | *phoU* | PA5365 |
| 9.89 | probable chemotaxis protein methyltransferase |  | PA3348 |
| 5.21 | ribose-phosphate pyrophosphokinase | *prs* | PA4670 |
| 1.24 | probable methyltransferase |  | PA1459 |
| 3.38 | probable chemotaxis transducer |  | PA1608 |
| 12.01 | phosphoribosylamine-glycine ligase | *purD* | PA4855 |
| 2.33 | chemotactic transducer PctA | *pctA* | PA4309 |
| 13.34 | chemotactic transducer PctC | *pctC* | PA4307 |
| 6.40 | probable acyl-CoA dehydrogenase |  | PA4979 |
| 9.15 | two-component sensor NtrB |  | PA5124 |
| 25.57 | lipopolysaccharide biosynthetic protein LpxO2 | *lpxO2* | PA0936 |
| 1.22 | phosphoenolpyruvate synthase | *ppsA* | PA1770 |
| 3.00 | probable chemotaxis transducer |  | PA1930 |
| 9.93 | probable enoyl-CoA hydratase /isomerase |  | PA1240 |
| 3.32 | exotoxin A precursor | *toxA* | PA1148 |
| 1.81 | probable enoyl-CoA hydratase/isomerase |  | PA0745 |
| 3.25 | probable acyl-CoA dehydrogenase |  | PA0746 |
| 3.90 | probable acyl-CoA dehydrogenase |  | PA0507 |
| 2.16 | probable FAD-dependent mono-oxygenase |  | PA2587 |
| 3.75 | probable two-component response regulator |  | PA1335 |
| 8.58 | phosphoribosylaminoimidazole synthetase | *purM* | PA0945 |
| 2.46 | glycine-cleavage system protein T1 | *gcvT1* | PA5215 |
| 17.76 | glucose-6-phosphate 1-dehydrogenase | *zwf* | PA3183 |
| 3.84 | molybdopterin biosynthetic protein A1 | *moaA1* | PA3870 |
| 3.72 | molybdopterin biosynthetic protein A2 | *moaA2* | PA1505 |
| 9.44 | molybdopterin biosynthetic protein C | *moaC* | PA3918 |
| 5.50 | membrane protein component of ABC phosphate | *pstA* | PA5367 |
| 3.31 | probable ATP-binding component of ABC |  | PA4223 |
| 2.69 | catalase | *katA* | PA4236 |
| 4.40 | catalase | *katB* | PA4613 |
| 1.24 | probable lyase |  | PA3517 |
| 1.63 | enolase | *eno* | PA3635 |
| 1.13 | probable two-component sensor |  | PA3044 |
| 16.05 | probable two component sensor |  | PA3078 |
| 9.13 | probable cystathionine gamma-lyase |  | PA0400 |
| 8.12 | HxcV putative pseudopilin |  | PA0680 |
| 8.27 | HxcU putative pseudopiln |  | PA0678 |
| 55.45 | HxcX atypical pseudopilin |  | PA0682 |
| 4.32 | probable adenosine deaminase |  | PA0148 |
| 2.76 | probable cytochrome P450 |  | PA2475 |
| 157.33 | probable transcriptional regulator |  | PA0436 |
| 1.14 | fatty-acid oxidation complex alpha-subunit | *faoA* | PA3014 |
| 1.62 | probable purine-binding chemotaxis protein |  | PA1464 |
| 1.02 | two-component response regulator PhoB | *phoB* | PA5360 |
| 1.85 | acetyl-CoA carboxylase beta subunit | *accD* | PA3112 |
| 1.96 | probable permease of ABC transporter |  | PA1340 |
| 3.43 | probable glycosyl transferase |  | PA5000 |
| 1.48 | ribulose-phosphate 3-epimerase | *rpe* | PA0607 |
| 2.58 | probable enoyl-CoA hydratase /isomerase |  | PA4980 |
| 2.94 | phosphoglycerate kinase | *pgk* | PA0552 |
| 3.10 | probable two-component sensor |  | PA3271 |
| 3.12 | uroporphyrinogen decarboxylase |  | PA5034 |
| 2.61 | probable acetolactate synthase large subunit |  | PA4977 |
| 2.32 | probable type II secretion system protein |  | PA4304 |
| 11.15 | negative regulator for alginate biosynthesis | *mucB* | PA0764 |
| 3.13 | positive regulator for alginate biosynthesis | *mucC* | PA0765 |
| 6.11 | serine protease MucD precursor | *mucD* | PA0766 |
| 1.81 | ferripyoverdine receptor | *fpvA* | PA2398 |
| 4.91 | sigma factor RpoS | *rpoS* | PA3622 |
| 2.67 | tRNA (guanine-N1)-methyl transferase | *trmD* | PA3743 |
| 1.02 | long-chain-fatty-acid-CoA ligase | *fadD1* | PA3299 |
| 7.01 | thiol:disulfide interchange protein DsbG | *dsbG* | PA2476 |
| 3.24 | phosphoribosylaminoimidazole synthetase | *purN* | PA0944 |
| 1.14 | alkaline metalloproteinase precursor | *aprA* | PA1249 |
| 6.99 | phosphoribosylformylglycinamidine synthase | *purL* | PA3763 |
| 4.48 | glycerol kinase | *glpK* | PA3582 |
| 4.94 | probable chemotaxis transducer |  | PA4915 |
| 4.07 | tRNA pseudouridine 55 synthase | *truB* | PA4742 |
| 1.44 | signal recognition particle receptor FtsY | *ftsY* | PA0373 |
| 28.46 | probable short-chain dehydrogenase |  | PA1470 |
| 2.09 | type III secretory apparatus protein PcrD | *pcrD* | PA1703 |
| 5.23 | secretion protein SecA | *secA* | PA4403 |
| 1.47 | adenine phosphoribosyltransferase | *apt* | PA1543 |
| 19.42 | chorismate synthase | *aroC* | PA1681 |
| 1.45 | probable flagellar assembly protein |  | PA1103 |
| 8.69 | probable acyl-CoA dehydrogenase |  | PA2552 |
| 2.49 | probable porin |  | PA0755 |
| 9.45 | probable two-component sensor |  | PA4886 |
| 4.89 | probable acyl-CoA dehydrogenase |  | PA1020 |
| 36.57 | probable trans-membrane sensor | *fpvR* | PA2388 |
| 10.67 | polyribonucleotide nucleotidyl transferase |  | PA4740 |
| 3.78 | probable two-component response regulator |  | PA3948 |
| 2.21 | probable two-component sensor |  | PA2810 |
| 5.50 | delta 2-isopentenylpyrophosphate transferase | *miaA* | PA4945 |
| 6.39 | probable two-component sensor |  | PA2480 |
| 13.94 | probable enoyl-CoA hydratase /isomerase |  | PA2841 |
| 1.76 | probable non-ribosomal peptide synthetase |  | PA2402 |
| 1.25 | arginine decarboxylase (ADC) | *speA* | PA4389 |
| 5.45 | methionyl-tRNA synthetase | *metG* | PA3482 |
| 13.63 | probable trans-membrane sensor | *foxR* | PA2467 |
| 10.12 | probable TonB-dependent receptor | *foxa* | PA2466 |
| 17.56 | gamma-carboxygeranoyl-CoA hydratase, GnyH | *gnyH* | PA2013 |
| 5.96 | quorum-sensing control repressor | *qscR* | PA1898 |
| 2.28 | aspartate ammonia-lyase | *aspA* | PA5429 |
| 4.82 | probable transcriptional regulator |  | PA2885 |
| 3.87 | histidine ammonia-lyase | *hutH* | PA5098 |
| 4.53 | thiol peroxidase | *tpx* | PA2532 |
| 6.51 | probable acyl-CoA dehydrogenase |  | PA0506 |
| 2.82 | probable type II secretion system protein |  | PA0686 |
| 1.88 | probable two-component sensor |  | PA2656 |
| 4.82 | probable cold-shock protein |  | PA0456 |
| 32.89 | probable cold-shock protein |  | PA0961 |
| 6.47 | carbamoylphosphate synthetase large subunit | *carB* | PA4756 |
| 1.34 | transcriptional regulator ToxR | *toxR* | PA0707 |
| 9.48 | probable acyl-CoA dehydrogenase |  | PA2889 |
| 4.30 | probable peptidyl-prolyl cis-trans isomerase |  | PA3871 |
| 10.99 | probable two-component response regulator |  | PA1437 |
| 14.97 | PcpS | *pcpS* | PA1165 |
| 5.54 | glutaminyl-tRNA synthetase | *glnS* | PA1794 |
| 11.52 | GMP synthase | *guaA* | PA3769 |
| 8.47 | adenylate cyclase | *cyaA* | PA5272 |
| 5.44 | probable histidine/phenylalanine ammonia-lyase |  | PA5093 |
| 2.04 | signal recognition particle protein Ffh | *ffh* | PA3746 |
| 3.42 | DNA mismatch repair protein MicA | *micA* | PA0382 |
| 2.87 | glutaminase-asparaginase | *ansB* | PA1337 |
| 8.83 | probable acyl-CoA dehydrogenase |  | PA0879 |
| 3.72 | peptide methionine sulfoxide reductase | *msrA* | PA5018 |
| 7.40 | carbamate kinase | *arcC* | PA5173 |
| 3.00 | ribosome recycling factor | *frr* | PA3653 |
| 2.30 | probable acyl-CoA dehydrogenase |  | PA5020 |
| 2.85 | isoleucyl-tRNA synthetase | *ileS* | PA4560 |
| 3.50 | response regulator GacA | *gacA* | PA2586 |
| 6.25 | Transcriptional regulator | *mvfR* | PA1003 |
| 3.30 | hypothetical protein, iron-regulated |  | PA2465 |
| 6.95 | conserved hypothetical protein, amino acid transport and metabolism |  | PA0006 |
| 1.29 | hypothetical protein, Protection and adaptation |  | PA4775 |
| 7.37 | conserved hypothetical protein, amino acid transport and metabolism |  | PA1638 |
| 4.36 | hypothetical protein, signal transduction mechanisms |  | PA0847 |
| 12.32 | hypothetical protein, signal transduction mechanisms |  | PA5487 |
| 3.86 | hypothetical protein, motility & attachment |  | PA4300 |
| 2.43 | hypothetical protein, signal transduction mechanisms |  | PA0431 |
| 2.78 | conserved hypothetical protein, transcription regulator |  | PA0512 |
| 1.46 | conserved hypothetical protein, membrane protein |  | PA0752 |
| 4.14 | hypothetical protein, , motility and chemotaxis |  | PA1463 |
| 17.05 | hypothetical protein, membrane component and transporters |  | PA2658 |
| 2.11 | hypothetical protein, motility and attachment | *fppA* | PA4295 |
| 6.42 | conserved hypothetical protein, cell motility |  | PA1442 |
| 2.81 | hypothetical protein, iron- regulated |  | PA4471 |
| 6.12 | conserved hypothetical protein, translation |  | PA4673 |
| 7.40 | hypothetical protein, iron-regulated membrane protein |  | PA0435 |
| 12.18 | hypothetical protein, lipid transport and metabolism |  | PA3286 |
| 8.00 | hypothetical protein, amino acid transport and metabolism |  | PA4774 |
| 18.08 | conserved hypothetical protein, iron-regulated |  | PA0637 |
| 1.81 | conserved hypothetical protein, nucleotide transport and metabolism |  | PA0387 |
| 7.90 | hypothetical protein, membrane protein |  | PA0434 |
| 10.51 | conserved hypothetical protein, membrane protein |  | PA0751 |
| 14.32 | hypothetical protein, iron- regulated |  | PA0615 |
| 7.65 | amino acid transport and metabolism | *pauA3* | PA1566 |
| 10.92 | hypothetical protein, amino acid transport and metabolism | *pauA5* | PA3356 |
| 10.10 | hypothetical protein, signal transduction mechanisms |  | PA4332 |
| 4.94 | Lipid transport and metabolism | *desB* | PA4888 |
| 4.00 | Nucleotide transport and metabolism | *nrdD* | PA1920 |
| 1.37 | hypothetical protein, iron- regulated |  | PA0616 |
| 5.04 | motility & attachment | *fimX* | PA4959 |
| 7.70 | hypothetical protein, iron- regulated |  | PA4467 |
| 3.41 | hypothetical protein, lipid transport and metabolism |  | PA0098 |
| 2.75 | hypothetical protein, energy production and conversion |  | PA4978 |
| 1.75 | conserved hypothetical protein, Cell wall/membrane/envelope biogenesis |  | PA4457 |
| 63.20 | motility and attachment | *tadZ* | PA4303 |
| 2.82 | conserved hypothetical protein, signal transduction mechanisms |  | PA1107 |
| 2.58 | hypothetical protein, cell motility |  | PA1095 |
| 3.14 | hypothetical protein, Intracellular trafficking, secretion, and vesicular transport | *tadG* | PA4294 |
| 2.70 | hypothetical protein, Energy production and conversion |  | PA4978 |
| 16.80 | hypothetical protein, Antibiotic resistance and susceptibility |  | PA1874 |
| 3.90 | conserved hypothetical protein |  | PA2971 |
| 3.40 | conserved hypothetical protein |  | PA5492 |
| 5.90 | hypothetical protein |  | PA1167 |
| 10.30 | hypothetical protein |  | PA4884 |
| 15.10 | conserved hypothetical protein |  | PA2412 |
| 19.90 | conserved hypothetical protein |  | PA0935 |
| 12.90 | hypothetical protein |  | PA4202 |
| 7.02 | hypothetical protein |  | PA4299 |
| 1.50 | conserved hypothetical protein |  | PA4562 |
| 7.90 | hypothetical protein |  | PA2464 |
| 2.90 | hypothetical protein |  | PA0433 |
| 2.90 | hypothetical protein |  | PA2689 |
| 54.15 | hypothetical protein hypothetical protein PA0754 |  | PA0754 |
| 19.89 | conserved hypothetical protein |  | PA0581 |
| 3.16 | hypothetical protein |  | PA2659 |
| 1.57 | hypothetical protein |  | PA3762 |
