# Supplementary material for: Dissection of the cis-2-decenoic acid signaling network in Pseudomonas aeruginosa using microarray technique
Source: Front Microbiol. 2015 Apr 28;6:383. doi: 10.3389/fmicb.2015.00383 (PMC4412052; doi:10.3389/fmicb.2015.00383)
Supplement: Supplementary file 3 [file Table3.DOCX]

**Supplemental Table 3.** Genes down-regulated by CDA in *P. aeruginosa* PAO1 biofilm determined through microarray analysis. The ratio of gene expression in CDA treated biofilm cells to that of untreated biofilm cells (fold down-regulation) is displayed for genes that exhibited changes >1-fold. Ratios are the average of three independent experiments.

| Fold changes | Function or class | Gene | ORF or operon |
| --- | --- | --- | --- |
| 2.24 | copper resistance protein B precursor | *pcoB* | PA2064 |
| 2.11 | usher CupA3 | *cupA3* | PA2130 |
| 1.67 | fimbrial subunit CupA4 | *cupA4* | PA2131 |
| 1.52 | chaperone CupB2 | *cupB2* | PA4085 |
| 1.60 | adhesive protein CupB5 | *cupB5* | PA4082 |
| 2.28 | probable aldolase |  | PA3430 |
| 1.58 | probable acetolactate synthase large subunit |  | PA4180 |
| 1.57 | probable chemotaxis transducer, Chemotaxis |  | PA4633 |
| 1.62 | probable transposase |  | PA4797 |
| 1.86 | probable DNA methylase |  | PA1678 |
| 1.64 | probable ATP-binding component of ABC |  | PA2812 |
| 1.68 | choline dehydrogenase | *betA* | PA5372 |
| 1.71 | choline sulfatase | *betC* | PA0031 |
| 1.50 | cytoplasmic axial filament protein | *cafA* | PA4477 |
| 1.53 | probable rubredoxin reductase | *rubB* | PA5349 |
| 1.64 | probable major facilitator superfamily (MFS) |  | PA2701 |
| 1.53 | probable transcriptional regulator |  | PA2766 |
| 1.55 | probable major facilitator superfamily (MFS) |  | PA2092 |
| 1.61 | D-alanine-D-alanine ligase A | *ddlA* | PA4201 |
| 1.11 | hypothetical protein | *pelA* | PA3064 |
| 1.77 | hypothetical protein | *pelB* | PA3063 |
| 1.75 | electron transfer flavoprotein beta-subunit, | *etfB* | PA2952 |
| 1.66 | arginine/ornithine binding protein AotJ | *aotJ* | PA0888 |
| 1.72 | type 4 fimbrial biogenesis protein PilO | *pilO* | PA5042 |
| 1.65 | probable two-component response regulator |  | PA2572 |
| 1.51 | xanthine dehydrogenase, Nucleotide transport and metabolism | *xdhA* | PA1524 |
| 1.60 | probable oxidoreductase |  | PA3256 |
| 1.51 | probable asparagine synthetase |  | PA2084 |
| 1.52 | benzoylformate decarboxylase | *mdlC* | PA4901 |
| 1.67 | probable chemotaxis transducer |  | PA2867 |
| 1.55 | adenosine diphosphate sugar pyrophosphatase | *aspP* | PA4971 |
| 1.89 | probable biotin-requiring enzyme, Lipid transport and metabolism |  | PA0493 |
| 1.92 | probable transcriptional regulator |  | PA2432 |
| 1.69 | phosphoglycerate mutase, Carbohydrate transport and metabolism | *pgm* | PA5131 |
| 1.84 | lipoate-protein ligase B | *lipB* | PA3997 |
| 1.74 | probable pseudouridylate synthase |  | PA0733 |
| 1.59 | undecaprenyl pyrophosphate synthetase | *uppS* | PA3652 |
| 1.60 | phosphomethylpyrimidine kinase, | *thiD* | PA3975 |
| 1.59 | probable decarboxylase |  | PA2108 |
| 1.78 | probable ATP-binding component of ABC |  | PA3538 |
| 2.03 | dTDP-4-dehydrorhamnose 3,5-epimerase | *rmlC* | PA5164 |
| 1.51 | Holliday junction DNA helicase RuvB, DNA | *ruvB* | PA0967 |
| 1.73 | 3-guanidinopropionase | *gpuA* | PA0288 |
| 1.57 | Dihydroorotase, Nucleotide transport and metabolism | *pyrQ* | PA5541 |
| 1.71 | probable choline transporter |  | PA5291 |
| 1.78 | probable ATP-dependent RNA helicase |  | PA2840 |
| 1.79 | d-erythro-7,8-dihydroneopterin triphosphate | *folX* | PA3439 |
| 1.57 | still frameshift 3-PHOSPHOSHIKIMATE |  | PA3164 |
| 1.7 | malonate decarboxylase alpha subunit | *mdcA* | PA0208 |
| 1.51 | probable ferredoxin |  | PA3491 |
| 1.60 | probable transcriptional regulator |  | PA1399 |
| 1.89 | transport protein ExbB | *exbB1* | PA0198 |
| 1.79 | probable nucleoside phosphorylase |  | PA3004 |
| 1.62 | probable transcriptional regulator |  | PA1201 |
| 1.57 | probable ATP-binding component of ABC |  | PA3376 |
| 1.95 | cell division inhibitor MinD | *minD* | PA3244 |
| 1.72 | probable 3-hydroxyisobutyrate, dehydrogenase |  | PA1576 |
| 1.85 | CsaA protein | *csaA* | PA3221 |
| 1.59 | thiazole biosynthesis protein ThiI | *thiI* | PA5118 |
| 1.91 | phenylalanine-4-hydroxylase | *phhA* | PA0872 |
| 1.57 | probable ferredoxin |  | PA4772 |
| 1.53 | cobalamin (5'-phosphate) synthase, | *cobV* | PA1281 |
| 1.76 | L-aspartate oxidase | *nadB* | PA0761 |
| 1.66 | probable permease of ABC transporter |  | PA3671 |
| 1.63 | precorrin-6y-dependent methyltransferase CobL | *cobL* | PA2907 |
| 1.86 | probable O-antigen acetylase |  | PA5238 |
| 1.53 | probable acyl-CoA dehydrogenase |  | PA1535 |
| 2.93 | phosphomannomutase AlgC | *algC* | PA5322 |
| 1.54 | GDP-mannose 6-dehydrogenase AlgD | *algD* | PA3540 |
| 1.65 | alginate-c5-mannuronan-epimerase AlgG | *algG* | PA3545 |
| 1.64 | probable biotin carboxylase/biotin carboxyl |  | PA2891 |
| 1.50 | probable glutathione S-transferase, |  | PA0473 |
| 1.65 | multidrug resistance protein |  | PA5159 |
| 1.62 | polyhydroxyalkanoate synthesis protein PhaF | *phaF* | PA5060 |
| 1.72 | probable acyl-CoA thiolase |  | PA3589 |
| 1.50 | Phosphoribosyl aminoimidazole carboxamide | *purH* | PA4854 |
| 1.50 | regulatory protein NosR | *nosR* | PA3391 |
| 1.55 | probable glycosyl transferasel |  | PA1390 |
| 1.68 | cytochrome b561 |  | PA0918 |
| 1.60 | probable hydroxamate-type ferrisiderophore | *fiuA* | PA0470 |
| 1.51 | probable biotin-dependent carboxylase | *atuC* | PA2888 |
| 1.89 | ribonuclease D | *rnd* | PA1294 |
| 1.58 | probable short-chain dehydrogenase |  | PA4089 |
| 1.97 | probable ATP-binding component of ABC |  | PA3212 |
| 1.59 | probable ATP-binding component of ABC |  | PA2350 |
| 1.70 | probable permease of ABC branched chain amino acid |  | PA4912 |
| 1.68 | 4-hydroxybenzoate-octaprenyl transferase, respiration | *ubiA* | PA5358 |
| 1.81 | probable transcriptional regulator |  | PA1859 |
| 1.72 | probable class III pyridoxal phosphate-dependent |  | PA0530 |
| 1.78 | probable transcriptional regulator |  | PA5403 |
| 2.00 | probable aldolase |  | PA3430 |
| 1.89 | chitinase | *chiC* | PA2300 |
| 1.37 | fumarate hydratase | *fumC1* | PA4470 |
| 1.59 | hypothetical protein |  | PA2829 |
| 1.63 | hypothetical protein |  | PA0982 |
| 1.73 | conserved hypothetical protein |  | PA5237 |
| 1.50 | hypothetical protein |  | PA0338 |
| 1.56 | hypothetical protein |  | PA2842 |
| 1.66 | hypothetical protein |  | PA1096 |
| 1.57 | conserved hypothetical protein |  | PA0979 |
| 1.63 | hypothetical protein |  | PA1149 |
| 1.83 | hypothetical protein |  | PA4404 |
| 1.56 | conserved hypothetical protein |  | PA4510 |
| 1.94 | conserved hypothetical protein |  | PA4399 |
| 1.59 | hypothetical protein |  | PA3219 |
| 1.66 | hypothetical protein |  | PA0333 |
| 1.59 | hypothetical protein |  | PA1571 |
| 1.68 | hypothetical protein |  | PA1925 |
| 1.51 | conserved hypothetical protein |  | PA5536 |
| 1.61 | hypothetical protein |  | PA4048 |
| 1.78 | conserved hypothetical protein |  | PA3070 |
| 1.59 | hypothetical protein |  | PA3216 |
| 1.62 | hypothetical protein |  | PA1367 |
| 1.65 | hypothetical protein |  | PA0089 |
| 1.69 | hypothetical protein |  | PA3869 |
| 1.65 | hypothetical protein |  | PA2506 |
| 1.75 | hypothetical protein |  | PA4298 |
| 1.62 | hypothetical protein |  | PA4405 |
| 1.91 | hypothetical protein |  | PA3772 |
| 1.77 | conserved hypothetical protein |  | PA4149 |
| 1.55 | conserved hypothetical protein |  | PA2651 |
| 1.87 | hypothetical protein |  | PA2137 |
| 1.49 | hypothetical protein |  | PA0429 |
| 1.58 | hypothetical protein |  | PA2958 |
| 1.52 | hypothetical protein |  | PA2189 |
| 1.54 | hypothetical protein |  | PA0193 |
| 1.61 | conserved hypothetical protein |  | PA4395 |
| 1.60 | hypothetical protein |  | PA2780 |
| 1.71 | hypothetical protein |  | PA1814 |
| 1.50 | hypothetical protein |  | PA3229 |
| 1.80 | hypothetical protein |  | PA0939 |
| 1.55 | conserved hypothetical protein |  | PA2771 |
| 2.08 | hypothetical protein |  | PA3402 |
| 1.77 | hypothetical protein |  | PA3143 |
| 1.73 | conserved hypothetical protein |  | PA4962 |
| 1.78 | hypothetical protein |  | PA0841 |
| 1.60 | hypothetical protein |  | PA3962 |
| 1.65 | hypothetical protein |  | PA5463 |
| 1.69 | hypothetical protein |  | PA1016 |
| 1.97 | hypothetical protein |  | PA3793 |
| 1.81 | hypothetical protein |  | PA3273 |
